# Electrochemical Sensors Based on the Electropolymerized Natural Phenolic Antioxidants and Their Analytical Application

**DOI:** 10.3390/s21248385

**Published:** 2021-12-15

**Authors:** Guzel Ziyatdinova, Ekaterina Guss, Elvira Yakupova

**Affiliations:** Analytical Chemistry Department, Kazan Federal University, Kremleyevskaya 18, 420008 Kazan, Russia; online2525@mail.ru (E.G.); elviraeakupova96@mail.ru (E.Y.)

**Keywords:** electrochemical sensors, electropolymerization, voltammetry, carbon nanomaterials, natural phenolics, antioxidants

## Abstract

The design and fabrication of novel electrochemical sensors with high analytical and operational characteristics are one of the sustainable trends in modern analytical chemistry. Polymeric film formation by the electropolymerization of suitable monomers is one of the methods of sensors fabrication. Among a wide range of the substances able to polymerize, the phenolic ones are of theoretical and practical interest. The attention is focused on the sensors based on the electropolymerized natural phenolic antioxidants and their analytical application. The typical electropolymerization reaction schemes are discussed. Phenol electropolymerization leads to insulating coverage formation. Therefore, a combination of electropolymerized natural phenolic antioxidants and carbon nanomaterials as modifiers is of special interest. Carbon nanomaterials provide conductivity and a high working surface area of the electrode, while the polymeric film properties affect the selectivity and sensitivity of the sensor response for the target analyte or the group of structurally related compounds. The possibility of guided changes in the electrochemical response for the improvement of target compounds’ analytical characteristics has appeared. The analytical capabilities of sensors based on electropolymerized natural phenolic antioxidants and their future development in this field are discussed.

## 1. Introduction

The development and application of chemical sensors are common in modern electroanalysis. Different types of modifiers, such as carbon nanomaterials, metal, metal oxide, and other compounds, such as nanoparticles, metal–organic frameworks, ionic liquids, surfactants, and polymeric materials, are actively investigated. Polymeric coverage formed by electrochemical polymerization is one of the attractive ways of sensor surface modification. This approach allows for the formation of polymeric films with minimal use of additional chemical reagents and volatile organic solvents that agree with the “green chemistry” concept. Furthermore, the polymeric coverage structure and thickness can be easily controlled by setting electrochemical process parameters. In comparison to chemical methods, electropolymerization provides a simplicity and rapidity of polymer synthesis, as well as stability.

The controlled electrochemical synthesis of polymeric films can be realized by three approaches, i.e., galvanostatic, potentiostatic, and potentiodynamic [1]. In galvanostatic and potentiostatic modes, a constant current or potential is applied to the working electrode for a certain time, on which the polymeric coverage thickness depends. Electropolymerization in the potentiodynamic mode is based on the multiple potential cycling in the iven range. In this case, the thickness of coverage formed depends on the number of potential cycles. The potentiodynamic mode of electropolymerization is the most often used method [1].

The properties of the coverage obtained are caused by the monomer nature allowing for the formation of conductive and non-conductive polymers [1]. The most typical examples of monomers undergoing oxidative polymerization are aniline, pyrrole, thiophene, and thiazine derivatives that form conducting polymeric films [2,3,4] that are well-known in the electroanalysis of a wide range of substances. On the other hand, phenol and aminophenol electropolymerization provides the insulating coverage formation [5,6,7]. Due to the small thickness of such coatings (10–100 nm), the target analytes and their oxidation/reduction products can easily diffuse to/from the modified electrode surface, decreasing its response time and improving the selectivity of analyte determination. In contrast to conducting films, phenol-based insulating polymers show low capacitive currents that significantly improve the shape of target analytes’ voltammograms and the accuracy of their determination. These advantages have been successfully used in electrochemical sensor fabrication [8,9,10]. Moreover, such films can reduce the matrix effects and prevent the contamination of the electrode surface with the analytes’ reduction/oxidation products.

Thus, electropolymerized phenols as a sensitive layer of the sensors and the application of this type of sensor in electroanalysis are of theoretical and practical interest. The current review is focused on the fabrication aspects of the sensors based on the electropolymerized natural phenolic antioxidants and their analytical capabilities. Special attention is paid to the sensors based on the combination of carbon nanomaterials with electropolymerized natural phenolics and their application in organic electroanalysis. Both the possibility of the synthesis of molecularly imprinted polymers (MIPs) based on electropolymerized natural phenolic antioxidants and an application for the detection of proteins and low-molecular organic compounds are discussed.

## 2. Electropolymerization of Phenolic Compounds as a Route for the Sensor Surface Modification

### 2.1. Electropolymerization of Phenols and Aminophenols

Phenol electropolymerization can be successfully realized on the metal- (Fe, Cu, Ni, Ti, Au, Pt) and carbon-based electrodes (glassy carbon (GCE), carbon paste (CPE), graphite electrodes) [11,12]. The process is usually performed in the basic medium that simplifies electron detachment from phenols existing as phenolate ions, though there are several examples of electropolymerizations of *o*-chloro- and *o*-hydroxy-substituted phenols in 0.6 M of sulphuric acid [13]. However, the electrode surface is gradually inactivated irrespectively of the medium pH. Such an effect is caused by the formation of an insoluble polymeric coverage leading to the electrode surface blockage [14].

**Scheme 1:**
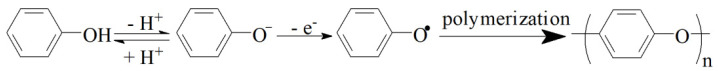
Phenol electropolymerization. Reprinted from [8] with permission from Elsevier.

The phenol forms a phenoxyl radical during electropolymerization. It can undergo oxidation to quinone or an addition reaction in *o*- and *p*-positions with the formation of oligomers and polymers (Scheme 1) that, consequently, give poly(phenylene oxide) film [8].

The relative rate of the oxidation process and polymerization depends on different factors; in particular, the monomer concentration, electrode material, electrolysis parameters, nature, and pH of the supporting electrolyte, as well as additional reagents used [7,14].

The electropolymerization of phenol derivatives (for example, 3-nitrophenol, pyrogallol, 4-hydroxybenzenesulfonic acid, and bromothymol blue) proceeds in a similar way [8]. The electropolymerization of coniferyl alcohol on the platinum electrode in 0.2 M NaOH leads to the formation of a dehydrogenation polymer called artificial lignin [15]. The reaction proceeds without the formation of a dimer in the solution that confirms electropolymerization at the electrode surface. Such behavior is similar to the in situ biosynthesis of lignin.

**Scheme 2:**
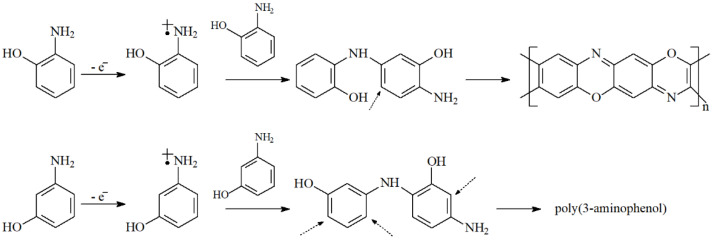
Aminophenol electropolymerization in acidic medium. Adapted from [17,18].

Aminophenols also undergo electropolymerization with the formation of insulating polymeric films [9,10,16]. The mechanism depends on the supporting electrolyte pH. Thus, the amino group participates in the polymerization process in an acidic medium (Scheme 2) [17,18].

**Scheme 3:**
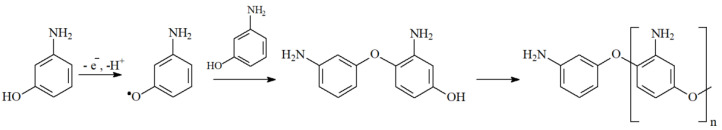
Electropolymerization of 3-aminophenol in neutral and basic medium.

Polymeric chain growth is driven by phenoxyl radicals in the neutral and basic medium [19]. The typical scheme is presented in Scheme 3 with the example of 3-aminophenol.

### 2.2. Electropolymerization of Natural Phenolic Antioxidants

**Figure 1:**
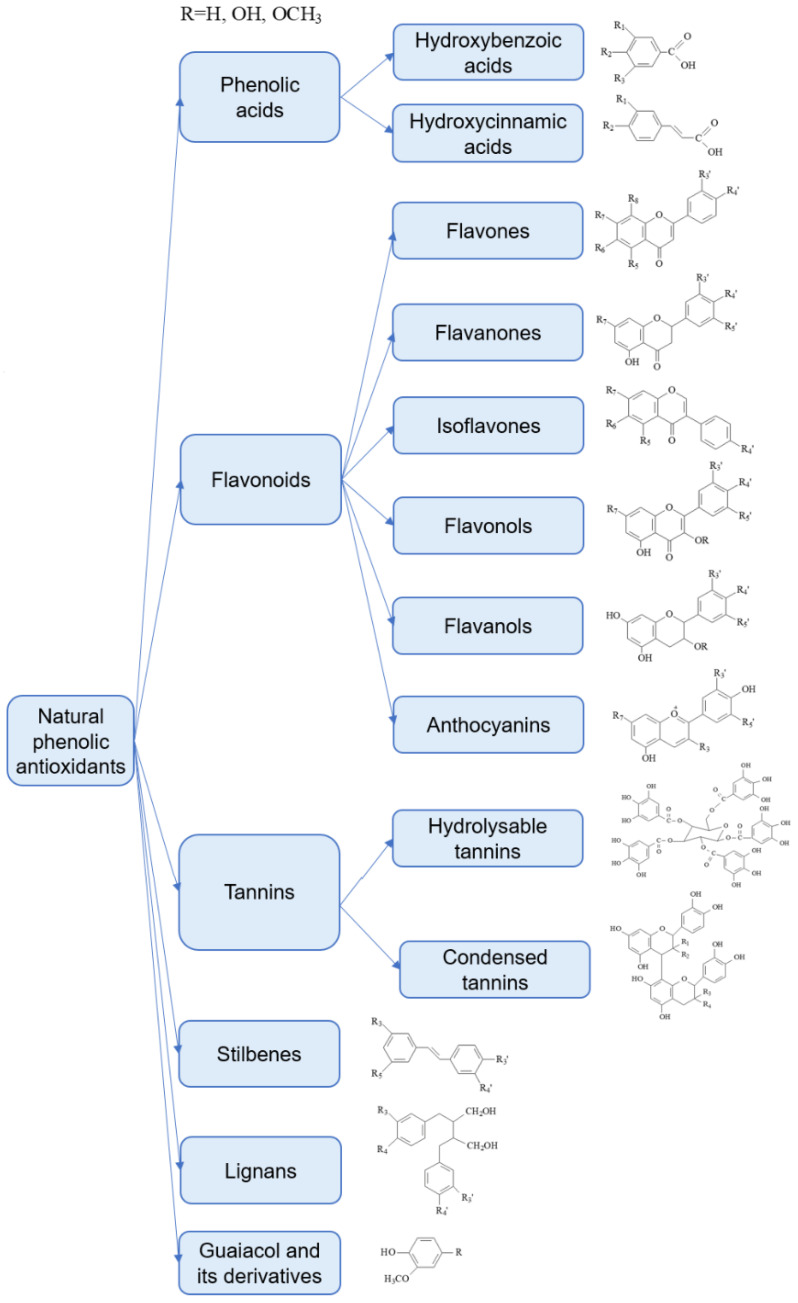
Classification of the natural phenolic antioxidants.

Among a wide range of phenolic compounds, natural phenolic antioxidants of plant origin can be considered as prospective ones due to their electrochemical activity and ability to electropolymerize. Natural phenolic antioxidants classification is presented in Figure 1. Their major classes are phenolic acids (hydroxybenzoic and hydroxycinnamic acids), flavonoids, tannins, stilbenes, lignans, and guaiacol and its derivatives. Flavonoids are the most representative ones thus far, as more than ten thousand compounds are known to date [20]. Other classes show a smaller variety of compounds. Therefore, a wide range of natural phenolic antioxidants opens novel horizons in the field of electrochemical sensors due to their bio- and structural diversity [20]. Nevertheless, the number of investigations in the field of electropolymerized natural phenolics is incomparable with electrochemically synthesized conducting polymers and some non-conducting polymers.

**Scheme 4:**
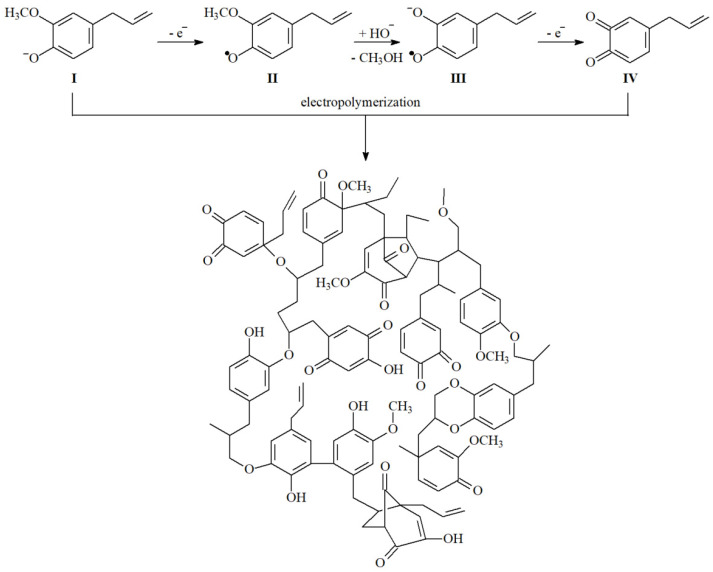
Eugenol oxidation and hypothesized structure of polyeugenol. Adapted from [21] with permission from the American Chemical Society.

Eugenol (4-allyl-2-methoxyphenol) is the most studied natural phenol in the context of electrochemical polymerization. Its voltammetric behavior has been studied in detail at the platinum [21,22], GCE [23,24], gold [24,25], and titanium [26] electrodes in a basic medium under conditions of potentiodynamic electrolysis. Like phenol, eugenol exists as a phenolate ion that is easily oxidized with phenoxyl radical II formation. Then, the basic hydrolysis leads to anion radical III, which consequently undergoes one-electron oxidation to *o*-quinone (Scheme 4) [21].

The electropolymerization process can involve several types of interactions [21]:

- Phenoxyl radical polymerization with poly(oxyphenylene) film [27];
- Diels–Alder reaction between IV and double bond forming 1,4-benzenedioxane fragment [21];
- Dimerization of phenoxyl radicals, and oxidative coupling of I with the formation of lignan-like structures [28];
- Hydroxylation of IV giving *p*-quinone fragments [29].

The polyeugenol film structure has been confirmed by Fourier transform infrared spectroscopy and is presented in Scheme 4 [21].

**Scheme 5:**
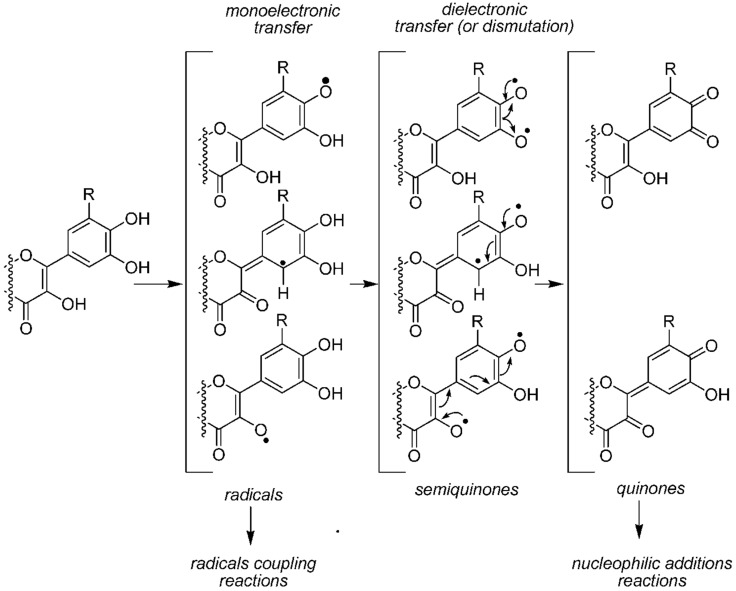
Oxidation pathway of flavonoids. Reprinted from [30].

Flavonoid electropolymerization is also based on the formation of phenoxyl radicals in the ring B due to the one-electron oxidation or semiquinone radicals via two-electron oxidation or dismutation (Scheme 5) [30,31]. Then, the radical coupling and oxidation to quinones can take place. The quinones can undergo nucleophilic addition reactions.

Phenolic acids electropolymerization occurs in two possible ways:

- Via phenoxyl radical formation and its following reactions;
- Via a conjugated double bond.

**Scheme 6:**
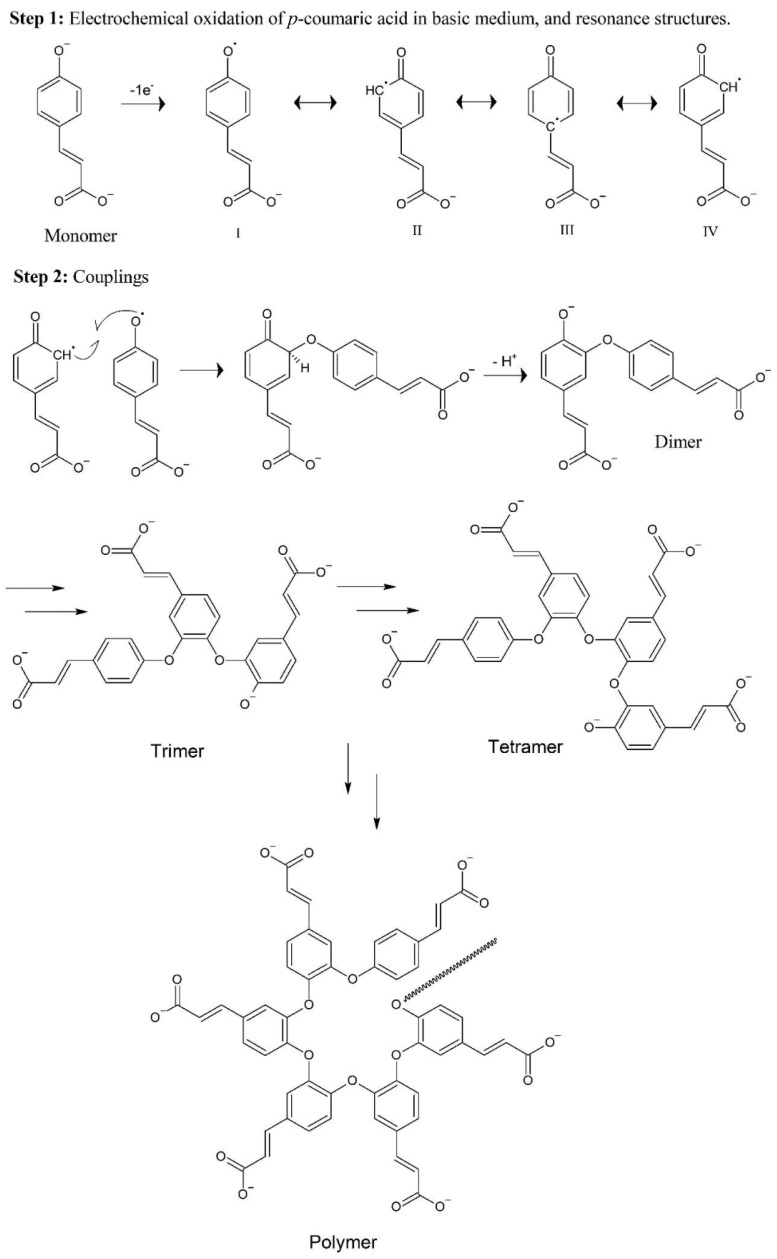
Electropolymerization of *p*-coumaric acid in basic medium. Reprinted from [37] with permission from Elsevier.

The first one is realized in the case of hydroxybenzoic acids [32,33]. Both mechanisms mentioned above can proceed in the case of hydroxycinnamic acids [34,35]. In general, demethylation, methoxy group addition, and radical coupling reactions occurred at the electrode surface during electropolymerization [36]. The electropolymerization of *p*-coumaric acid in the basic medium is mainly realized via phenoxyl radical formation and coupling reactions, as Scheme 6 shows [37].

The electropolymerization of natural phenolic antioxidants is successfully achieved in both potentiodynamic and potentiostatic modes, whereas the galvanostatic electrodeposition of polymeric films is not reported to date. Typical examples and corresponding conditions of potentiodynamic electropolymerization are presented in Table 1.

**Figure 2:**
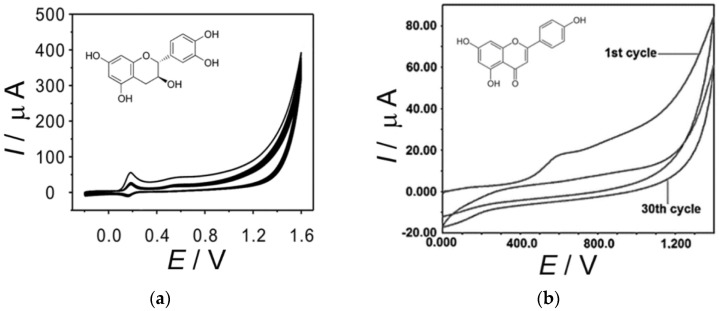
(**a**) Cyclic voltammograms of 1.0 mM catechin at activated CPE in 0.1 M phosphate buffer pH 7.4. Reprinted with modification from [44] with permission from the Royal Society of Chemistry; (**b**) cyclic voltammograms of 1.0 mM apigenin at GCE in 1:4 methanol−phosphate buffer mixture pH 7. Reprinted with modification from [47] with permission from the Royal Society of Chemistry.

Typical cyclic voltammograms of natural phenolic antioxidant electropolymerization are presented in Figure 2 with examples of catechin [44] and apigenin [47] given. A gradual decrease in the phenolic antioxidant oxidation currents is observed with the increase in the potential cycling, which indicates the formation of non-conducting polymeric coverage. The full disappearance of the peak is observed in the case of monophenols in ring B (Figure 2b), in contrast to polyphenolics with *o*-dihydroxybenzene fragments (Figure 2a).

**Figure 3:**
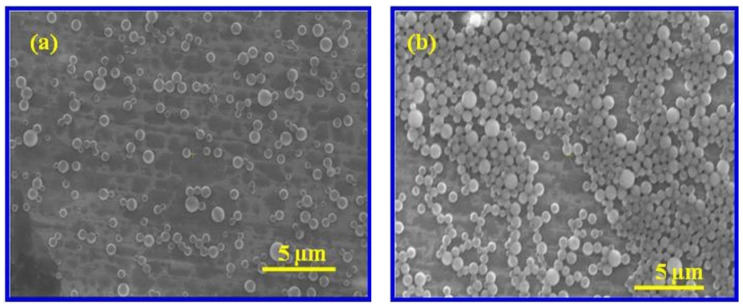
SEM images of curcumin drop-casted on the electrode before electropolymerization (**a**) and polycurcumin formed after 20 cycles of potentiodynamic electrolysis (**b**). Reprinted from [48] with permission from Elsevier.

Recently, the coverage of polycurcumin nanospheres has been obtained by potentiodynamic electrolysis. Curcumin has been preliminary adsorbed at the electrode surface and dried under vacuum conditions. Then, the electropolymerization has been performed in 0.1 M phosphate buffer (pH 7.0) for 20 cycles from −0.3 V to 0.8 V at the potential scan rate of 50 mV s^−1^ [48]. The electropolymerization leads to the conjugation of micron-scale curcumin molecules and the formation of elongated structures with branched chains that form nanosphere-shaped polymeric film that is evenly distributed at the electrode surface (Figure 3). The average diameter of the polycurcumin particles is 250 nm.

The potentiostatic mode of electropolymerization is seldom used. It can be performed as electrolysis for a given time at a certain potential or using chronoamperometry. The electrodeposition of poly(caffeic acid) on the surface of GCE has been successfully achieved by 30 s electrolysis at 2.0 V in phosphate buffer pH 7.0 from 0.2 mM caffeic acid [49,50,51]. Gallic acid electropolymerization has been carried out at 1.0 V for 60 s [52]. The chronoamperometry of 1.0 mM rutin at 1.4 V for 40 min in a phosphate buffer of pH 7.0 has been applied for the polyrutin-modified paraffin-impregnated graphite electrode fabrication [38]. The polyrutin layer consists of irregular islands 7–10 μm in size and an overlap accumulation film of 50–120 nm thickness due to the rapid reaction. For comparison, the potentiodynamic mode with the same electrolysis time and potential window of 0.0–1.4 V gives more uniform coverage of high porosity, with cavities of 10–300 nm. These data indirectly explain the seldom usage of the potentiostatic mode for the creation of electropolymerized films based on natural phenolic antioxidants. Furthermore, potential cycling allows for a better control of the electropolymerization process using changes in the monomer oxidation peak on the cyclic voltammograms.

A combination of potentiodynamic and potentiostatic electropolymerization has been applied for the preparation of over-oxidized polymeric films. In this case, the electropolymerization is performed in the potentiodynamic mode, whereas its further over-oxidation in the basic medium is carried out using chronoamperometry. This methodology has been demonstrated with an example of rutin electropolymerization [53]. Over-oxidation leads to the formation of homogenous bud-like particles and, as a result, provides an increase in the electrode surface area.

The electropolymerization conditions are varied depending on the monomer (Table 1). Thus, a strongly basic medium is used for eugenol, vanillin, and tannin electropolymerization. Neutral and weakly acidic media are applied for flavonoids that are caused by their acid-base properties (pKa for flavonoids is in the range of 6.0–11.5 [54]). In general, the electropolymerization conditions have to be optimized depending on the analyte to which quantification the sensor fabricated. The analyte response is significantly changed with the variation of electrolysis parameters and the supporting electrolyte nature and pH, as well as the monomer concentration [32,35,55]. The number of cycles (or electrolysis time) and monomer concentration strongly affect the polymeric coverage properties as far as determining its thickness. Taking into account the insulating properties of electropolymerized phenols, a high monomer concentration and a number of cycles (or electrolysis time) can lead to a decrease in the electron transfer rate, or even a full blockage. Nevertheless, the multi-factor optimization of electropolymerization conditions is often out of consideration, which is probably caused by the tedious and time-consuming experiments required.

**Figure 4:**
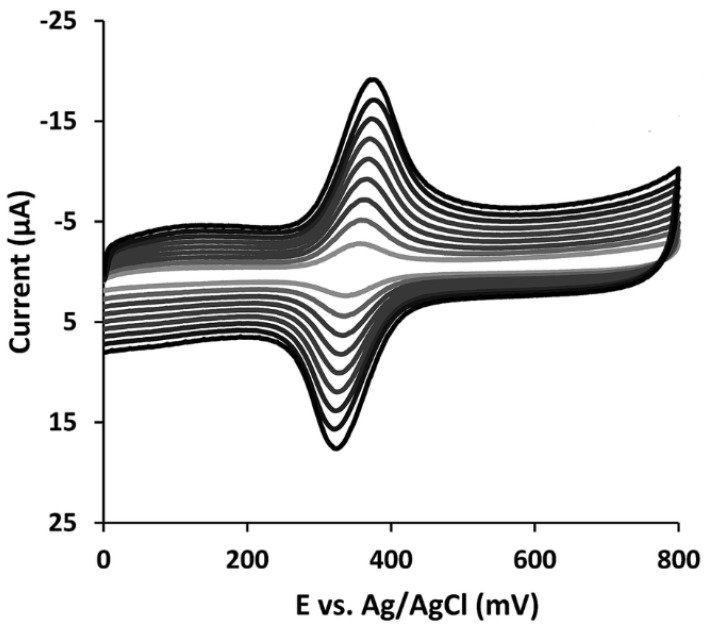
Cyclic voltammograms of the poly(caffeic acid)/GCE in 0.05 M phosphate buffer pH 4.8 at various potential scan rates (25, 50, 75, 100, 125, 150, 175, 200, 225, 250 mV s^−1^). Reproduced with modification from [51] with permission from the Royal Society of Chemistry.

Polymeric coverages based on the natural phenolic antioxidants, excluding monophenols (such as *p*-coumaric acid [35,37] or naringin [55]), are electrochemically active due to the presence of *o*-quinone/catechol fragments in their structure. Their reduction/oxidation leads to the appearance of reversible redox steps in the cyclic voltammograms [21,22,23,24,25,26,31,32,33,34,37,38,43,44,45,46,48,49,50,51,56] (Figure 4).

**Scheme 7:**
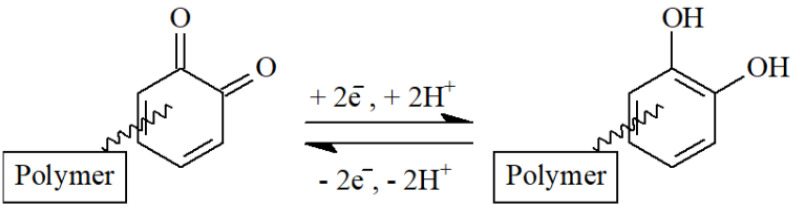
Reduction of *o*-quinone fragments in the natural phenol-based polymer.

Redox peaks potentials and currents are pH-dependent as long as the electrochemical process occurs with proton participation (Scheme 7). Such behavior of the electrodes suggests that their action is on an electrocatalysis basis, which is widely used in electroanalytical chemistry at the moment. This topic is discussed in detail in review [31].

Sensors based on the electropolymerized phenolic antioxidants have shown wide analytical capabilities that are discussed in the following sections.

## 3. Analytical Application of the Sensors Based on the Electropolymerized Natural Phenolics

Bare carbon- and metal-based electrodes modified with electropolymerized natural phenolic antioxidants of different classes have shown applicability in both organic and inorganic electroanalysis.

### 3.1. Application in Organic Analysis

Neuromediators (dopamine, epinephrine, and serotonin) and several other bioactive compounds, such as ascorbic acid and sulfur-containing antioxidants, are the major analytes in the organic analysis. A lot of attention is paid to the simultaneous determination of neuromediators, with uric and ascorbic acids being a typical problem in biological fluids analysis. For example, the simultaneous quantification of dopamine and ascorbic acid on the polyvanillin-modified CPE [39] and poly(caffeic acid)/GCE [49] has been shown. The selective detection of dopamine in the presence of ascorbic acid has been achieved in the polyeugenol-based sensor [21,23]. Polyvanillin-modified CPE provides a simultaneous determination of epinephrine and uric acid [41]. A simultaneous determination of dopamine and serotonin in a polycatechin-based sensor [44], as well as epinephrine, serotonin, and ascorbic acid in a polyrutin-modified electrode [38], are important progresses in the field. The analytical characteristics of neuromediator determination are presented in Table 2.

The sensor based on the graphite electrode with polyeugenol film has been used for the determination of hydroquinone in cosmetics [57]. The electrocatalytic nature of the analytical signal has been proved. Under conditions of cyclic voltammetry in a phosphate buffer of pH 7.0, the linear dynamic ranges are 1.42–101.07 and 136.67–480.50 μM of hydroquinone, with the detection and quantification limits of 1.44 and 4.37 μM, respectively.

Acidified by sulfuric acid, GCE with coverage of poly(caffeic acid) acts as a sensor for the metanephrine *O*-methylated extraneuronal metabolite of catecholamines, which is considered to be a biomarker of phaeochromocytoma [58]. The sensor gives a linear response in the range of 0.5–40 μM, with a detection limit of 0.17 μM in the linear sweep voltammetry mode in 0.1 M HNO_3_. The analytical characteristics are significantly improved vs. other electrochemical sensors. Moreover, the high selectivity of the metanephrine response in the presence of 150-fold excesses of inorganic ions (K^+^, Na^+^, Fe^3+^, Ca^2+^, Mg^2+^, Cl^−^, CO_3_^2−^, and SO_4_^2−^) and 100-fold excesses of L-cysteine, tyrosine, glycine, phenol, uric acid, lactate, histidine, and glucose allows for the application of the sensor in biological fluid analyses, as has been shown for human serum.

**Figure 5:**
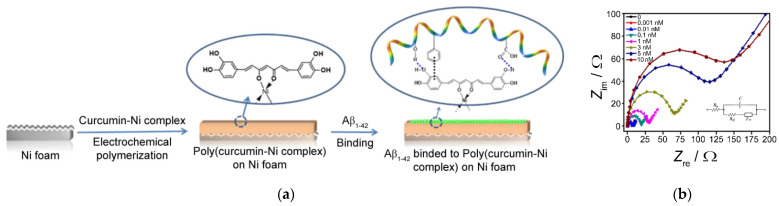
(**a**) Schematic representation of the synthesis of poly(curcumin-Ni complex) and its binding to amyloid β oligomer (Aβ_1–42_); (**b**) Nyquist plots of poly(curcumin-Ni complex) sensor after incubation in various concentrations of amyloid β oligomer solution. Reprinted from [59] with permission from Elsevier.

Another interesting sensor is described for the determination of amyloid β oligomer, which is considered to be a molecular biomarker for Alzheimer’s disease diagnostics. The application of the Ni foam substrate as a transducer and poly(curcumin-Ni) complex as a sensitive layer (Figure 5a) provides a highly sensitive impedimetric response to the amyloid β oligomer in the concentration range of 0.001–5.0 nM, with a detection limit of 0.001 nM (Figure 5b) [59]. A relatively simple preparation and stability, label-free direct detection of the amyloid β oligomer, and cost-effectivity are the advantages of this sensor.

Sulfur-containing compounds play an important biological function in living systems [60]. The ability to be oxidized on the electrode surface stipulated the active development of electrochemical sensors with the polymer-modified surface for the determination of such compounds. The voltammetric detection of L-cysteine in the presence of glutathione and DL-homocysteine has been demonstrated on the GCE modified with polyeugenol [24]. The excellent selectivity in the presence of structurally similar sulfur-containing compounds is caused by the pore size in the polymeric film providing adsorption of L-cysteine. Glutathione and DL-homocysteine, due to having bigger steric sizes, cannot penetrate the electrode surface. Another approach for the L-cysteine determination is based on the electrochemical impedance estimation of the polyeugenol-modified titanium electrode in the presence of an analyte [26]. The electron transfer resistance at the open circuit potential is increased proportionally to the L-cysteine concentration in the range of 10–100 μM.

A novel voltammetric approach for the determination of α-lipoic acid is based on its oxidation on poly(vanillin)-modified platinum electrode. The linear dynamic range of 0.03–2.00 mM has been obtained using adsorptive square-wave voltammetry in the Britton–Robinson buffer of pH 5.0. The limit of detection is 0.025 mM [61]. The analytical characteristics are not impressive and are even worse than reported for bare GCE [62]. Nevertheless, the method can be used for the quality control of α-lipoic acid pharmaceutical dosage forms containing high amounts of the active substance.

The poly(caffeic acid)-based sensor has shown an electrocatalytic response to acetaminophen and has been successfully applied for the pharmaceutical dosage form analysis [50]. Under conditions of square-wave voltammetry, the analytical ranges of 0.1–1.0 and 1.0–10 μM have been observed with the detection limit of 0.026 μM. The selectivity towards acetaminophen in the presence of ascorbic and uric acids, dopamine, and *p*-aminophenol is the main advantage of the sensor developed.

The co-polymerization of natural phenolics is of practical interest, although limited examples have been reported to date. Platinum electrode electrochemically modified with poly(vanillin-co-caffeic acid) has been presented for the quantification of curcumin in spices. The co-polymer deposition has been performed from a 6 mM mixture of monomers in a phosphate buffer of pH 7.4 by six-fold potential cycling from −0.80 to 1.0 V. The electrode has shown a linear response to curcumin in the ranges of 0.01–0.07 and 0.1–1.0 mM with the detection limit of 0.005 mM [63]. These characteristics are quite similar to those reported for bare GCE [64].

As can be concluded from the data of [61,63], the sensor surface modification with electropolymerized natural phenolic antioxidants does not mean a priori improvement of the analytical characteristics in comparison to bare electrodes. The correct choice of the monomer and analyte plays a crucial role in the successful results. In this case, the types of possible interactions of the analyte with the sensor surface (hydrophobic, including π-π stacking and electrostatic interactions, H-bonding or their complex, host–guest inclusion in the pores of polymeric film), taking into account the hydrophilic–lipophilic balance, solubility, and affinity of the analyte, have to be comprehensively considered.

**Figure 6:**
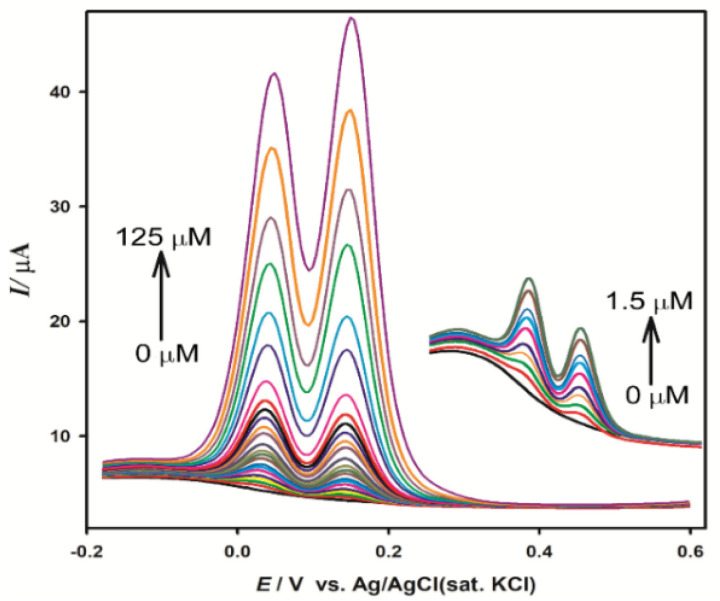
Differential pulse voltammograms of equimolar mixtures of hydroquinone and catechol at the over-oxidized polyrutin-based sensor in phosphate buffer pH 7.2. Reprinted from [53] with permission from Elsevier.

An over-oxidized polyrutin-based sensor has been applied for the highly sensitive simultaneous determination of hydroquinone and catechol [53]. The peak potential separation of 107 mV has been achieved in a phosphate buffer of pH 7.2 (Figure 6). The quantification of dihydroxybenzenes in differential pulse modes is possible in the ranges of 0.06–3.5 and 8.00–55 μM for both analytes, with detection limits of 0.031 and 0.053 μM for hydroquinone and catechol, respectively. The selectivity of the dihydroxybenzenes response in the presence of other phenolics (phenol, bisphenol A and equimolar resorcinol) makes the sensor applicable to tap and industrial wastewater analysis [53].

Sensors with electropolymerized natural phenolic antioxidants as a sensitive layer can be used as detectors in flow-injection analysis, which has been demonstrated with an example of polyeugenol-coated platinum electrodes [22]. A flow-injection system with a thin-layer dual-electrode amperometric detector consisting of GCE and polyeugenol/Pt has been successfully applied for the simultaneous quantification of ascorbic acid and hydrogen peroxide. The selectivity of the analyte response is caused by the difference in the electrode material. Ascorbic acid is oxidized on the GCE at 0.3 V. Hydrogen peroxide is selectively oxidized on the polyeugenol-modified electrode due to the size effects and the polymeric film properties providing an electrostatic repulsion of ascorbate ions. The method allows for the quantification of ascorbic acid and hydrogen peroxide in the range of 0.005–1.0 mM for both compounds, with the limits of detection equal to 1.0 and 0.50 μM, respectively. Some orange-taste soft drinks spiked with defined amounts of both analytes have been tested as a prototype of real samples. Besides its high sensitivity, the method is attractive due to the on-line and high-speed analysis using simple and cost-efficient equipment.

### 3.2. Application in Inorganic Analysis

Sensors based on the electropolymerized phenolic antioxidants have shown effectivity in inorganic electroanalysis. Heavy metal ions and nitric oxide are the most studied analytes.

**Figure 7:**
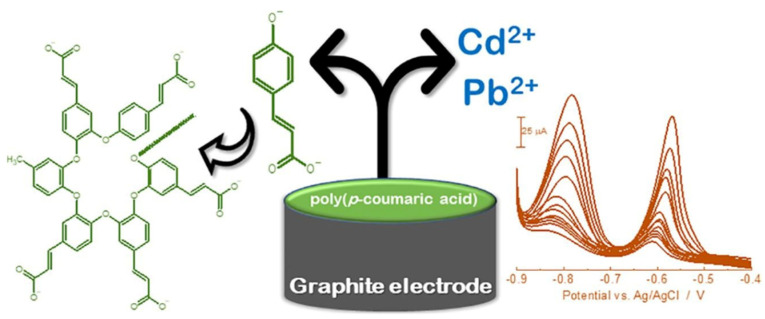
Poly(*p*-coumaric acid)-based sensor and its response towards the mixture of cadmium and lead in square-wave anodic stripping mode. Reprinted from [37] with permission from Elsevier.

The graphite electrode modified with poly(*p*-coumaric acid) has been applied for the simultaneous determination of cadmium and lead [37]. The sensor allows for heavy metal quantification under conditions of square-wave anodic stripping voltammetry in a 0.10 M acetate buffer pH of 4.50 (Figure 7). The linear dynamic range of 40–1000 ppb has been obtained for both analytes, with the detection limits of 15.4 and 13.6 ppb for Cd^2+^ and Pb^2+^, respectively. The sensor response is selective in the presence of other metal ions, excluding Cu^2+^. However, its interference effect can be overcome by masking with hexacyanoferrate(II) ions. Another advantage of the sensor is the stability of the response for three months. The analytical application of the sensor has been shown for spiked samples of freshwater and water.

GCE modified with poly(flavonoids) has been developed for the Cu(II) determination [45,46,47]. Polyluteolin-, polykaempferol- and polyapigenin-modified GCE have been obtained by potentiodynamic electrolysis. The electrode response is based on the Cu(II) complexation with the 5-hydroxy group in ring A and 4-carbonyl group in ring C of the flavonoids in the polymeric film structure. The complex formation has been achieved after 1 h for polyluteolin- and polykaempferol-based sensors and 2 h for polyapigenin/GCE. The electrodes have shown good reproducibility, stability, and selectivity towards Cu(II). Under conditions of cathodic differential pulse voltammetry, the linear dynamic range of 0.1–1000 nM Cu(II) with the detection limit of 10 pM have been obtained on both polyluteolin/GCE and polykaempferol/GCE [45]. The application of the square-wave mode allows for decreasing the Cu(II) detection limits to 6.0 pM on polyluteolin/GCE and 3.0 pM on polykaempferol/GCE, while the dynamic range is kept the same [46]. Polykaempferol/GCE shows a higher sensitivity than polyluteolin/GCE. The electrode surface regeneration can be easily performed by washing with ethylenediaminetetraacetic acid, forming stable complexes with Cu(II). Polyluteolin- and polykaempferol-based sensors have been used in tap water analysis. Polyapigenin/GCE has been applied for the Cu(II) quantification in soil under conditions of differential pulse voltammetry in a Britton–Robinson buffer of pH 5.0 [47]. The calibration graph is linear for 0.010–1000 nM, with a 10 pM detection limit. The absence of the interfering effect of 1000 nM Cd(II), Ni(II), Co(II), and Zn(II) on the analytical signal of Cu(II) has been proven.

A sensor with a nanosphere-shaped polycurcumin film has been utilized for the nanomolar quantification of Hg(II) in a real-time analysis of seawater [48]. The differential pulse anodic stripping voltammetry in a 0.1 M NH_4_NO_3_ solution (pH 5.5) has been used for the Hg(II) detection. A highly selective quantification in the range of 0.21–21.72 μg L^−1^ with the detection limit of 70.49 ng L^−1^ has been achieved.

Several approaches based on the polyeugenol-modified electrode have been developed for nitric oxide detection, with it being an important marker in biochemistry. The polyeugenol layer acts as a permselective membrane and provides selectivity and specificity toward NO^•^ by hydrophobic repulsion, ionic interaction, and molecular exclusion [23,65,66,67]. Nitrite oxide quantification is possible in the presence of peroxynitrite and hydrogen peroxide. The electrochemical sensor array for the simultaneous amperometric detection of nitric oxide and peroxynitrite has been developed [67]. The on-chip device is based on the sets of 110 gold ultramicroelectrodes with a 50 µm diameter, Ag/AgCl reference electrode, and gold counter electrode. Each working electrode of the network is covered with a thin layer of polyeugenol for the detection of nitric oxide. The amperometric detection of nitric oxide is carried out at 0.8 V in a phosphate buffer of pH 7.4. The network for the peroxynitrite detection has been based on bare gold electrodes operated at −0.1 V. The simultaneous detection of both analytes has been achieved using two amperometric systems acting together. Each of them consisted of seven gold ultramicroelectrodes (modified with polyeugenol for nitric oxide detection or bare for peroxynitrite).

A flow microsensor has been developed for a selective detection of the low micromolar concentration of nitric oxide in the presence of hydrogen peroxide and interfering species [68]. The device consisted of the working (polyeugenol-modified platinum) and integrated second platinum electrode (called the depletion electrode) within the microfluidic channel for the depletion of H_2_O_2_. Both electrodes were used simultaneously under constant flow conditions at 0.8 and 0.45 V, respectively. This system provides an upstream elimination of the interfering species by inducing their oxidation in the flowing solution before nitric oxide detection. The approach allows for the quantification of nitric oxide in the range of 0.2–6 μM with the detection limit of 0.08 μM.

**Figure 8:**
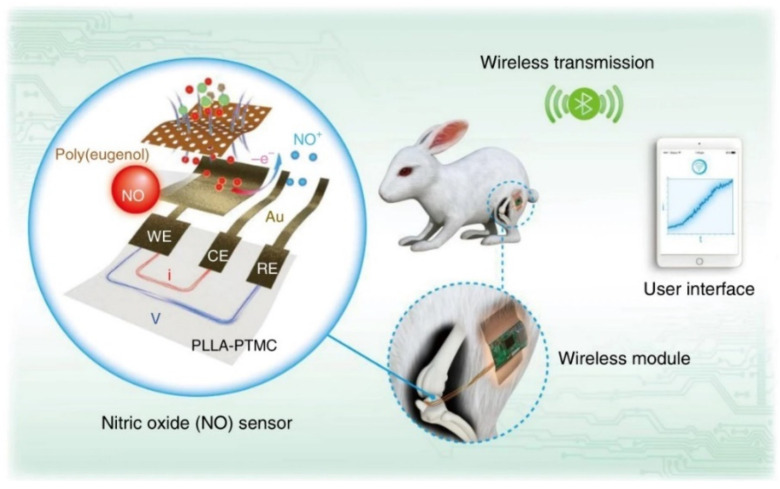
Schematic illustration of a transient NO sensor composed of a bioresorbable substrate (copolymer of poly(l-lactic acid) and poly(trimethylene carbonate)), Au nanomembrane electrodes, and a poly(eugenol) thin film. The sensor implanted in the joint cavity of a New Zealand white rabbit allows for continuously detecting NO concentrations in vivo and transmitting the data to a user interface through a customized wireless module. Reprinted from [69].

Further development in this field is demonstrated on the flexible and physically transient electrochemical sensor for real-time wireless nitric oxide monitoring (Figure 8).

The sensor consisted of a bioresorbable substrate (copolymer of poly(l-lactic acid) and poly(trimethylene carbonate)), and ultrathin gold nanomembrane electrodes covered with biocompatible poly(eugenol) film as the selective membrane [69]. The sensor shows a wide sensing range (0.01–100 μM), a low detection limit (3.97 nM), a fast response time (<350 ms), desirable stability (≥7 days), and excellent anti-interference characteristics. The real-time monitoring of nitric oxide has been demonstrated at the cellular and organ levels, as well as in vivo on the joint cavity of a rabbit for 5 days using a wireless data transmission system. The sensor has shown full degradation after 8 weeks of implantation in vivo without an apparent inflammatory or toxic effect.

As shown in the example of nitric oxide sensors, electropolymerized phenol-based sensors, in combination with microfluidic technologies, miniaturization, and multi-electrode arrays, make the selective and sensitive detection of highly reactive small molecules possible. Moreover, the application of wireless technologies opens new horizons for the in vivo real-time monitoring of biomarkers for fast and correct therapy.

The polyeugenol-based sensor is also proposed for the selective and sensitive detection of nitrite based on its preliminary chemical conversion to nitric monoxide in acidic aqueous solution using hexacyanoferrate(II) as a reduction agent [70]. An amperometric response at 0.9 V of a polyeugenol-modified rotating disc platinum electrode is linear in the range of 5.0–100 μM with the detection limit of 0.6 μM. The approach is tested on human saliva samples.

### 3.3. Electropolymerized Natural Phenolic Antioxidants as Protective Coatings

Films formed by electropolymerized eugenol [25] and isopropylmethylphenols [71,72] have been proposed as protective coatings. A gold electrode covered with a polyeugenol layer acts as a sensitive sensor for oxygen [25]. The polymeric film has a double function: the protection of the electrode material and provision of the antifouling effect. The sensor shows a sensitive response to the oxygen in the presence of human serum albumin, 5-hydroxytryptamine, and ascorbic acid, which is an important achievement that allows for sensor usage in biomedical analysis. The oxygen sensing in vitro in the range of 37.5–188 μM in the presence of artificial cerebral spinal fluid has been performed successfully. The approach can be considered as a promising tool in neuroscience and neurophysiology.

**Figure 9:**
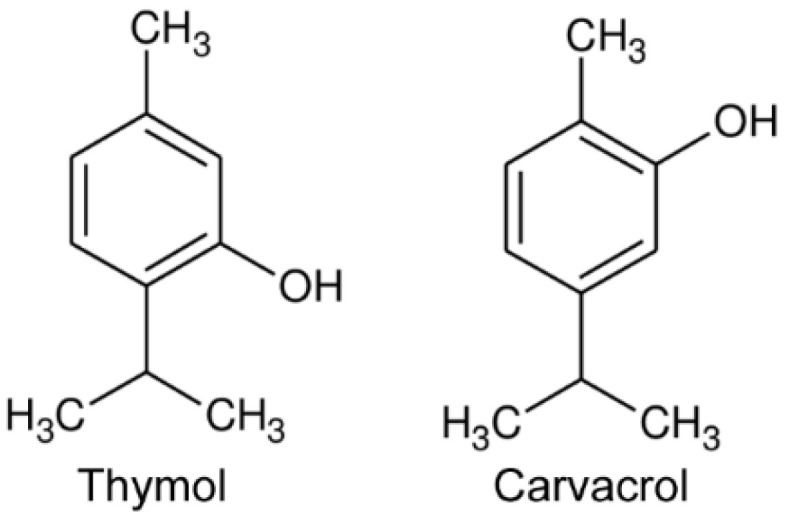
Isopropylmethylphenol structure.

Electropolymerization of isopropylmethylphenols (thymol and carvacrol (Figure 9)) has been performed by potentiodynamic electrolysis on the surface of copper electrode [71,72]. The morphology and properties of the polymeric coverage strongly depend on the monomer molecular structure. Thymol and carvacrol are isomers that differ by the position of the hydroxy group in the aromatic ring.

**Figure 10:**
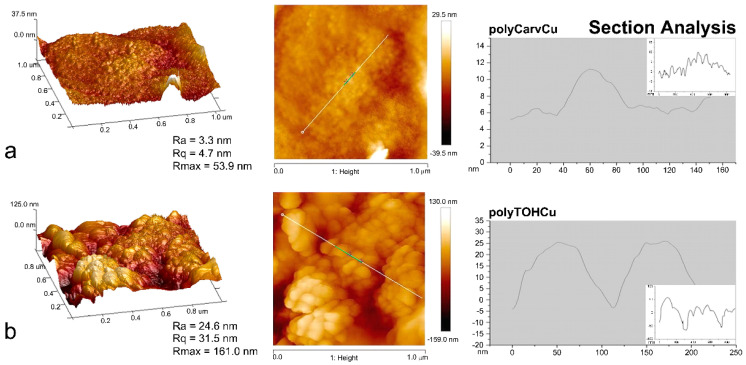
Atomic force microscopy images of polycarvacrol/Cu (**a**) and polythymol/Cu (**b**). Reprinted from [71] with permission from Elsevier.

The visual and morphological characteristics of polythymol and polycarvacrol have been obtained. The brownish layer consisted of globular particles close to 35 nm high forming cauliflower-like structures, and stick structures with cracks have been observed for the polythymol layer. The polycarvacrol film is transparent and formed by round nanoflakes that are less than 5 nm in height (Figure 10). The polymeric coverages obtained have been tested as anticorrosive ones. In contrast to polythymol, the polycarvacrol layer provides an anticorrosive defense of the copper electrode in 0.136 M KCl [71]. Further application of the sensor is shown on the identification of carvacrol in essential oils of *Origanum vulgare*, *Thymus vulgaris*, and *Origanum* × *applii* [72]. The approach is based on the electropolymerization of carvacrol on the copper electrode surface.

Summarizing the application aspects of bare electrodes with electropolymerized natural phenolics, it can be concluded that neuromediators, sulfur-containing compounds, heavy metals, and nitric oxide are the major analytes. The sensor response depends on the bare electrode material used, monomer structure, and electropolymerization conditions.

## 4. Sensors Based on the Combination of Nanomaterials and Electropolymerized Natural Phenolic Antioxidants and Their Analytical Capabilities

As mentioned above, the electropolymerization of phenolic compounds gives insulating films. Therefore, attention is paid to the conductivity of the sensitive layer, which can be enhanced by the combination of polymeric coverage with different types of conductive nanomaterials. This approach seems effective as long as nanomaterials provide sufficient conductivity and a high electrode surface area. The following electropolymerization of a suitable phenolic antioxidant provides a high loading and more uniform coverage of the electrode surface. The application of the polymeric film as a sensitive layer of the sensors allows for controlling the selectivity and sensitivity of the electrode response to the target analyte or group of structurally related compounds.

Carbon nanomaterials (nanotubes [32,33,34,35,52,55,56,73,74,75,76,77,78,79,80,81,82], carbon black [83,84], and graphene [85], including functionalized ones) are the most common conductive platform for the further electropolymerization of suitable phenolic monomers. Electrode surface modification is carried out using a layer-by-layer deposition. Carbon nanomaterials are usually prepared as suspensions in a suitable solvent and then drop-casted on the bare electrode surface. Natural phenolic electropolymerization is further performed in potentiodynamic or potentiostatic modes.

Recently, an original approach has been used for the in situ preparation of multi-walled carbon nanotubes (MWCNTs)-polyvanillin composite film [86]. Sweeping an applied bias at the carbon fiber microelectrode from −0.8 to 1.8 V at a scan rate of 50 mV s^−1^ for 25 cycles in aqueous solutions containing 2 mg mL^−1^ vanillin and 1 mg mL^−1^ MWCNTs in the absence of a supporting electrolyte provides a formation of the composite. This sensor gives an electrocatalytic response to nitrite in a wide range of concentrations (0.2–3100 µM) with a detection limit of 50 nM. The approach has been successfully applied to lake water analysis with satisfactory recovery. The composite of MWCNTs with poly(2-methoxy-4-vinylphenol) has been formed by in situ free radical polymerization using azobis(isobutyronitrile) as an initiator [87]. This approach provides polymer-grafted MWCNTs as both a method of functionalization and their entrapment in the polymeric layer. The homogeneity of the resulting composite nanomaterial is much higher than for a polymer composite obtained from the mixture of MWCNTs and polymer chains in the solution. The nanocomposite is stable for 6 months, after which, the electrochemical activation sensor shows a catechol/quinone redox couple response and provides an electrocatalytic effect to NADH. The amperometric NADH detection is possible in the range of 1–70 µM with the sensitivity of 7.5 nA µM^−1^ and detection limit of 0.29 µM.

As shown in the example of eugenol [56], the application of carbon nanomaterials as a platform for the electrodeposition of the polymer allows for decreasing the monomer concentration and the number of cycles required for electropolymerization. Moreover, the charge under the oxidation peak reflecting the amount (thickness) of the polymeric material deposited on the electrode surface is higher than for the bare electrode.

**Figure 11:**
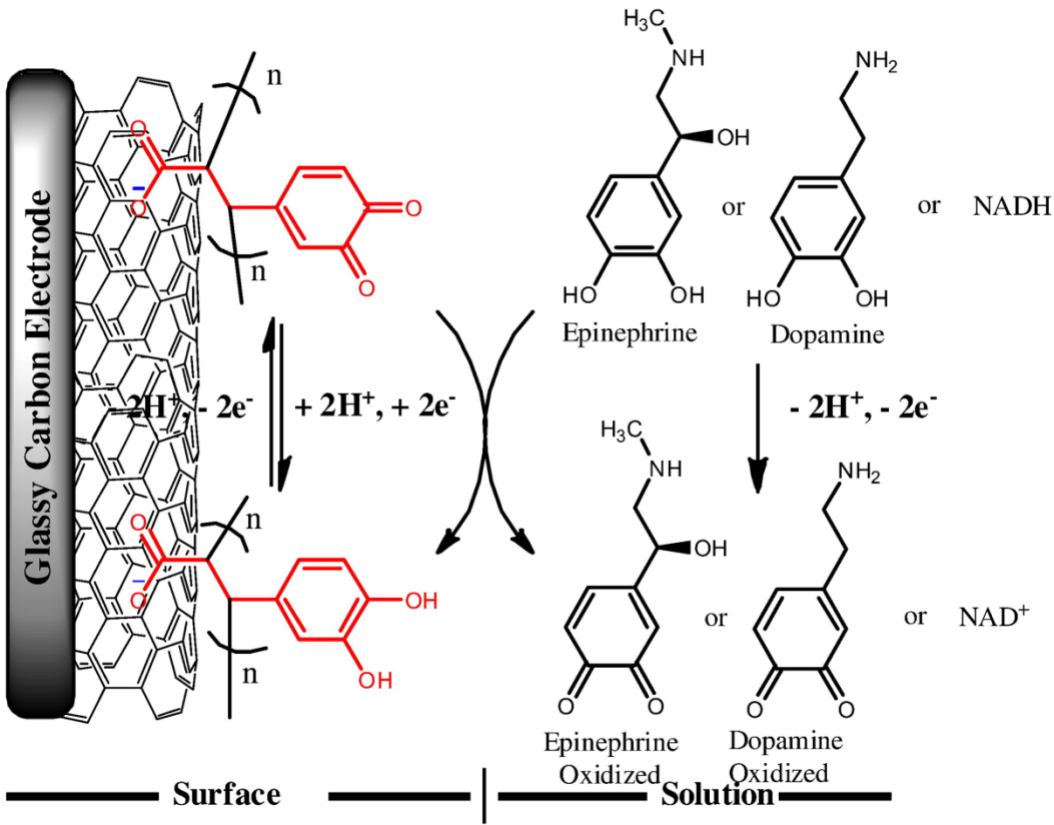
NADH, dopamine, and epinephrine oxidation scheme on poly(ferulic acid)/MWCNTs/GCE. Reprinted from [34] with permission from Elsevier.

Phenolic acids, flavonoids, curcumin, and guaiacol derivatives containing catechol fragments are the most often used among the monomers. Similar to the bare electrodes, the polymer formed on the carbon-nanomaterial-modified surface of the electrode is electrochemically active due to the redox pair quinone/catechol providing an electrocatalytic response of target analytes [31]. The scheme of the electrocatalytic response on such sensors is shown in the example of NADH, dopamine, or epinephrine for the poly(ferulic acid)/MWCNTs-based sensor (Figure 11).

The analytical application of the sensors based on the carbon nanomaterials and electropolymerized natural phenolic antioxidants is focused on the quantification of neuromediators [33,34,81], including a simultaneous determination with uric and ascorbic acids [33], NADH [34,83], sulfur-containing compounds (L-cysteine [35,84], and glutathione [82]), as well as natural phenolic antioxidants [32,52,56,73,74,75,76,77,78]. The analytical characteristics are summarized in Table 3. The sensors work under conditions of different types of voltammetry, amperometry, and chronomethods. Amperometry under stirred conditions has a higher current sensitivity than cyclic and can be comparable with differential pulse voltammetry. Furthermore, the stirring conditions provide less contamination of the sensor surface with the analyte oxidation products. On the other hand, the multi-component amperometric analysis [33] requires the subtraction of the currents at previous steps, which complicates the quantification procedure.

Chronomethods allow to improve the analytical characteristics of target analytes in comparison to differential pulse voltammetry, as shown in the examples of quercetin [32,73] and gallic acid [76,77,78].

The absence of the interfering effect of serotonin and uric acid on the voltammetric response of the poly(ferulic acid)/MWCNTs-based sensor towards NADH, dopamine, or epinephrine has been confirmed [34]. The interference of ascorbic acid can be eliminated by oxidation with ascorbate oxidase. However, other typical interferences, such as glucose, urea, norepinephrine, and L-DOPA, are out of consideration. A simultaneous determination of neuromediators is impossible. Thus, the insufficient selectivity of the sensor makes it applicable to the analysis of the monocomponent pharmaceutical dosage forms, which is a sufficient limitation [34]. On the contrary, a sensor based on poly(vanillic acid) can be used for the simultaneous quantification of dopamine and ascorbic and uric acids in human urine [33]. A layer-by-layer combination of carbon black with poly(caffeic acid) provides an electrocatalytic response towards NADH with a sensitivity of 0.007 µA µM^−1^ [83]. The fabrication of a flexible sensor based on single-walled carbon nanotubes obtained by filtration-press transfer methodology is the further development in this topic [88]. The electrode surface processed electrochemical carboxylation and functionalization with poly(caffeic acid). The device can be used both as an amperometric sensor and as a voltabsorptometric sensor for the NADH, depending on the analytical task.

The sensor based on carboxylated MWCNTs and electrodeposited polyquercetin has shown an electrocatalytic response towards the L-DOPA, uric acid, and tyramine. Under conditions of differential pulse voltammetry, the oxidation potentials of 156, 264, and 528 mV for the L-DOPA, uric acid, and tyramine, respectively, have been obtained, which allow for their simultaneous quantification. The selectivity of the sensor response in the presence of 0.1 M Mg^2+^ and Ca^2+^, 0.01 M L-lysine, L-asparagine, glycine, phenylalanine, *N*-acetyl-l-cysteine, glutathione, and L-cysteine allows for its application in the direct analysis of biological fluids [81].

Poly(*p*-coumaric acid)- [35] and poly(syringic acid)-based sensors [84] are characterized with an excellent selectivity towards L-cysteine in the presence of inorganic ions including nitrate and nitrite [84], typical interferences for biofluids (dopamine and ascorbic and uric acids [35,84], xanthine and hypoxanthine [84]) and other S-containing compounds (homocysteine, glutathione [35,84], L-methionine, L-cystine, and α-lipoic acid [35]), which is the main achievement allowing for an application of the sensors in clinical analysis. The high selectivity in the presence of other S-containing compounds is explained by the size effects based on scanning electron microscopy data. The pore size in the polymeric coverage allows for the penetration and retention of L-cysteine molecules via hydrophobic interactions [35]. On the contrary, the selectivity of glutathione detection using a sensor based on the “bamboo-like” carbon nanotubes and electropolymerized caffeic acid is fully out of consideration [82], although promising analytical characteristics have been obtained.

Another aspect of the analytical application of the sensors based on carbon nanomaterials and electropolymerized natural phenolic antioxidants is their sensitive response to phenolic compounds. Sensor based on the paraffin-wax-impregnated graphite electrode with mechanically immobilized MWCNTs and polycurcumin gives an electrocatalytic effect towards butylated hydroxyanisole. The preliminary drop-casting of 10 µL of 0.01 M curcumin on the electrode surface has been used. Then, the polymeric coverage has been obtained by 80-fold potential cycling with the scan rate of 50 mV s^−1^ from −0.4 to 0.6 V in the neutral medium [80]. Unfortunately, the selectivity study is not performed. Therefore, the applicability of the sensor to real samples is questionable.

**Figure 12:**
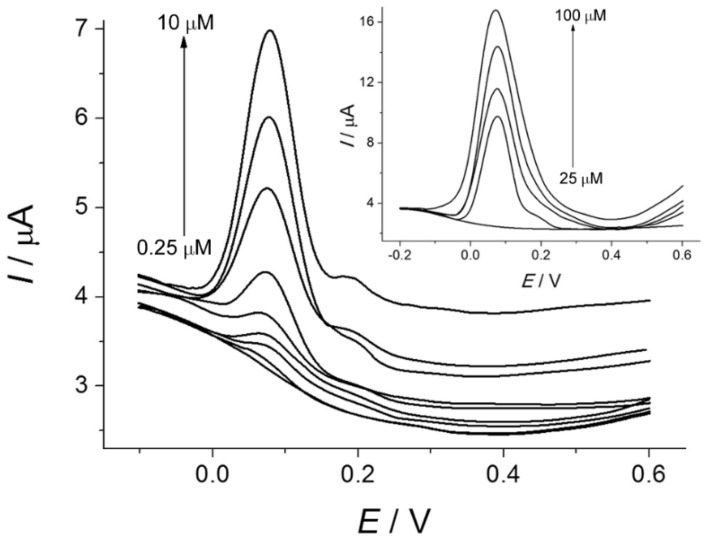
Differential pulse voltammograms of quercetin at the sensor based on MWCNTs and poly(gallic acid) in phosphate buffer pH 7.4. Reprinted from [32] with permission from Elsevier.

The presence of phenolic fragments in the structure of polymeric coverage provides a sensor response towards structurally related compounds; in particular, natural phenolics, including antioxidants [32,52,56,73,74,75,76,77,78]. A poly(gallic acid)-based sensor has been developed for the determination of quercetin [32] and capsaicinoids [74] in plant materials. The advantages of the approach for quercetin quantification are a high sensitivity at a physiological pH of 7.4 and a good shape of the voltammograms, even for the low oxidation currents (Figure 12), which excludes curve preprocessing (baseline correction or background subtraction). Moreover, the high selectivity of the quercetin response in the presence of other natural phenolics (rutin, vanillin, syringaldehyde, gallic, ferulic, *p*-coumaric, and sinapic acids) is achieved [32].

As for the capsaicinoids, a high sensitivity, wide linear dynamic ranges, and low detection limits have been obtained for the sensor based on MWCNTs and poly(gallic acid) in comparison to other chemically modified electrodes [74]. This is caused by hydrophobic interactions of aromatic rings in the polymeric film structure and capsaicinoids providing their accumulation at the sensor surface and increasing their determination sensitivity. The surface-controlled oxidation of capsaicinoids has been proved with the surface coverage of 33 ± 1, 22.4 ± 0.7, and 21.5 ± 0.7 pmol cm^−2^ for capsaicin, dihydrocapsaicin, and nonivamide, respectively [74]. Unfortunately, the oxidation potentials of capsaicinoids are the same, making their separate determination while coexisting impossible. On the other hand, such behavior can be considered as a group selectivity of the sensor response, allowing for the evaluation of total capsaicinoids contents in the sample, i.e., its pungency. The last one is an important parameter of the quality and safety of red hot pepper containing samples. The selectivity of the sensor towards capsaicinoids has been proven in the presence of a 1000-fold excess of inorganic ions (K^+^, Mg^2+^, Ca^2+^, NO_3_^−^, Cl^−^ SO_4_^2−^, and PO_4_^3−^) and saccharides (glucose, rhamnose, and sucrose), as well as carotenoids, α-tocopherol, and ascorbic acid being the most typical interfering substances for red-hot-pepper-based products. The sensor has been successfully applied for the analysis of red hot pepper spices and *Capsicum annuum* L. tinctures.

Among phenolic acids that have been used as monomers for the formation of polymeric coverage, ellagic acid (a lactonic form of gallic acid dimer) is of interest. A voltammetric sensor based on MWCNTs and electropolymerized ellagic acid has been fabricated by potentiodynamic electrolysis in the electrochemical window of 0.0–1.0 V at the potential scan rate of 100 mV s^−1^ in a phosphate buffer of pH 7.0 [75]. Seven cycles in a 10 µM monomer solution are enough to obtain a stable polymeric film consisting of evenly distributed 30–50 nm spherical particles. The sensor gives a highly sensitive response to naringin—a major phenolic antioxidant of grapefruit. The poly (ellagic acid) layer provides a preconcentration of naringin on the porous electrode surface via the hydrophobic interactions; in particular, the π-π staking of aromatic rings in naringin and polymer structures. An ultra-high selectivity to naringin in the presence of both typical interfering substances (inorganic ions, saccharides, and ascorbic acid) and other natural phenolics (gallic, ferulic, caffeic, chlorogenic acids, and hesperidin) is the favorable advantage of the sensor developed over other electrochemical methods. The applicability to real samples is successfully shown on the grapefruit juices, allowing for sensor usage for the fast screening of the juices as an alternative to more tedious and expensive chromatographic methods.

The structural similarity of the natural phenolic antioxidants leads to similar or close oxidation potentials. In this case, the total antioxidant parameters can be estimated for the complex samples, such as plant materials, foodstuff, beverages, etc. Thus, a sensor based on the MWCNTs and potentiostatically deposited poly(gallic acid) has been developed for the total phenolic contents determination [52]. Gallic acid has been used as a standard, with it being a typical antioxidant for foodstuff and plant materials. In order to improve the sensitivity of the response, the adsorptive anodic square-wave voltammetry with a 60 s accumulation time has been used. The approach is tested on pomegranate juice [52]. The accuracy of the voltametric approach is confirmed by the standard Folin–Ciocalteu method. Another poly(gallic acid)/MWCNTs-based sensor has been used for the evaluation of the antioxidant capacity of medicinal plant tinctures [73]. A poly(gallic acid) layer has been obtained by cyclic voltammetry in a phosphate buffer of pH 6.0 from a 10 μM monomer by 15 cycles in the potential window from −0.2 to 1.0 V at the scan rate of 100 mV s^−1^. The antioxidant capacity has been measured in quercetin equivalents using chronocoulometry at 1.0 V after 100 s electrolysis. It should be noted that the chronocoulometric mode of quercetin detection significantly improves its analytical parameters in comparison to a differential pulse mode [32].

Polyeugenol/MWCNTs/GCE has shown a group selectivity towards natural phenolics (phenolic acids, flavonoids, and tannin) oxidized at approx. 0.52 V in a differential pulse mode in 0.1 M HClO_4_. Therefore, a sensor has been applied for the estimation of the wine antioxidant capacity using catechin as a standard [56]. The approach is simple, does not require additional reagents, and gives reliable results. The small amount of the sample (50 or 25 µL for white and red wine, respectively) and direct analysis are also significant advantages of the method.

**Figure 13:**
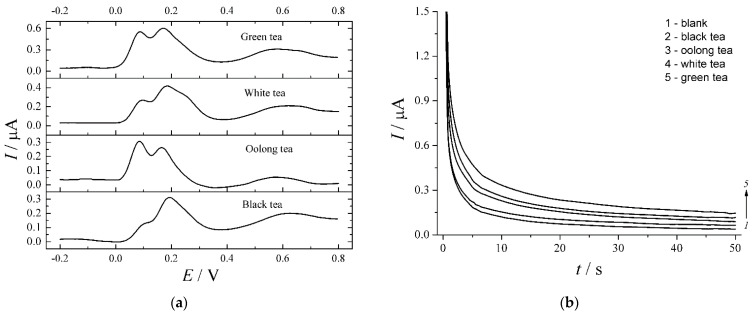
Typical baseline-corrected differential pulse voltammograms (**a**) and chronoamperograms (**b**) of tea on polyquercetin/MWCNTs/GCE in phosphate buffer pH 7.0. Reproduced with modification from [78] with permission from Springer Nature.

A sensor based on the combination of MWCNTs and polyquercetin has been successfully fabricated by potentiodynamic electrolysis in a basic medium [76,77,78]. The conditions of electropolymerization have been optimized in order to provide the best response of gallic acid, with it being a typical standard of natural phenolic antioxidants. Thus, the electropolymerization of quercetin has been performed from a 1.0 mM monomer solution by five-fold potential cycling from –0.1 to 1.0 V at the scan rate of 100 mV s^−1^ in 0.1 M NaOH. The sensor gives a highly sensitive response to gallic acid, catechin, and epigallocatechin gallate. Nevertheless, the similarity of the analyte oxidation potentials does not allow for their simultaneous determination. On the other hand, the sensor provides the possibility to measure the total contents of these phenolic antioxidants, which has been successfully realized on the tea samples. The antioxidant capacity of green, white, oolong, and black teas has been evaluated in the epigallocatechin gallate equivalents using differential pulse voltammetry [76] and in the gallic acid equivalents using chronoamperometry at 0.2 V [78]. Typical differential pulse voltammograms and chronoamperograms of tea samples are shown on Figure 13. Both methods have shown positive correlations with the standard ones (total phenolics contents and antioxidant activity towards 2,2-diphenyl-1-picrylhydrazyl). The same sensor has been applied for the estimation of polyphenol–protein interactions and their influence on the antioxidant capacity of tea [77]. The changes in the voltammetric response of tea polyphenols (catechin and epigallocatechin gallate) in the presence of milk proteins (casein, bovine serum albumin, and β-lactoglobulin) have proved the binding effect of proteins to polyphenols.

The main advantages of the polymer-based sensors developed for the total antioxidant parameter evaluation are their simplicity, rapidity, and reliability of response, as well as the usage of the own response of the sample, excluding the application of unstable reagents, such as 2,2-diphenyl-1-picrylhydrazyl, and the absence of limitations for the colored samples analysis.

Sensors based on carbon nanomaterials and electropolymerized curcumin can be used in inorganic analyses. GCE covered with MWCNTs and an electrodeposited curcumin layer consisting of grafted monomer molecules and a polymeric film shows electrocatalytic activity towards hydrazine oxidation. A reduced overpotential and an increased peak current in comparison to GCE and MWCNTs/GCE have been observed on the polymer-modified electrode [79]. The catalytic rate constant of 6.26 × 10^3^ M^−1^ s^−1^ has been found. The quantification of hydrazine is performed in an amperometric mode at 0.25 V in a 0.5 M phosphate buffer of pH 8.0. The sensitivity of the sensor response is 22.9 μA mM^−1^. The simplicity of fabrication, fast response, and good reproducibility are the main advantages of the sensor developed.

**Figure 14:**
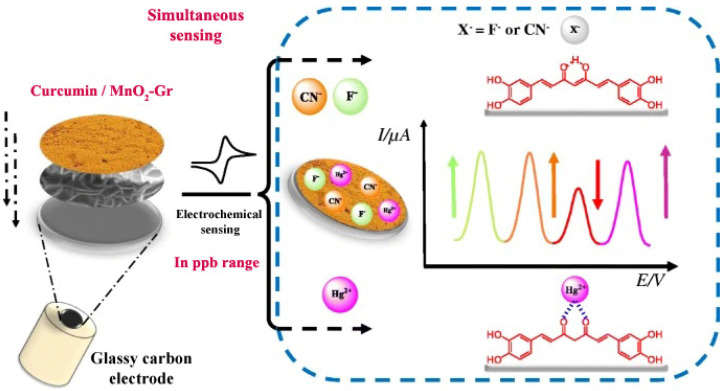
Schematic presentation of the polycurcumin-based sensor and its response to Hg(II), fluoride, and cyanide. Reprinted from [85] with permission from Springer Nature.

The layer-by-layer deposition of MnO_2_-graphene nanosheets and polycurcumin as a modifier of the GCE surface has been performed [85]. MnO_2_-graphene nanosheets improve the electron transfer rate and the electrical conductivity of the carbon surface. The polycurcumin layer has been electrodeposited by potentiodynamic electrolysis from the 50 μM curcumin solution in a phosphate buffer of pH 7.4 by 20 consecutive cycles from −0.3 to 0.6 V at the potential scan rate of 50 mV s^−1^. Polycurcumin acts as both an electrochemical transducer and an ion receptor. The sensor has shown a sensitive response to Hg(II), fluoride, and cyanide ions, including their simultaneous detection (Figure 14) at 0.82, −0.24, and 0.12 V, respectively. The ions under investigation participate in the complexation with electropolymerized curcumin at the electrode surface, which can be evaluated by differential pulse voltammetry and chronoamperometry. The simultaneous detection of cationic and anionic analytes is possible due to the keto-enolic tautomeric forms of the curcumin at the electrode surface. The keto form provides Hg(II) detection via O,O′-donor binding sites, while the hydroxyl group of the enol-form makes H-bonding with anions possible. The sensor gives a linear response in the range of 50–1200 ppb for all analytes, with the detection limits of 19.2, 17.2, and 28.3 nM for Hg(II), fluoride, and cyanide, respectively. An excellent selectivity of the sensor response to target ions in the presence of I^−^, CH_3_COO^−^, PO_4_^3−^, SCN^−^, and NO_3_^−^, as well as Mg^2+^, Fe^2+^, Zn^2+^, and Cu^2+^, is a significant achievement. Furthermore, the sensor shows storage stability for one month at 5 °C.

Thus, a combination of carbon nanomaterials with electropolymerized natural phenolic antioxidants as a sensitive layer of the electrochemical sensors shows wide analytical capabilities. Sensors show a high individual selectivity towards L-cysteine [35,84] and some neuromediators [33,34,35,36,37,38,39,40,41,42,43,44,45,46,47,48,49,50,51,52,53,54,55,56,57,58,59,60,61,62,63,64,65,66,67,68,69,70,71,72,73,74,75,76,77,78,79,80,81], allowing for their application in biomedical analysis. The group selectivity of sensors based on electropolymerized gallic acid, eugenol, and quercetin towards natural phenolics [32,52,56,73,74,75,76,77,78] can be successfully used in the quality control of plant-material-based samples and foodstuff using total antioxidant parameters (total phenolics contents and antioxidant capacity).

## 5. Electropolymerized Natural Phenolics as a Platform for Immobilization of Other Modifiers

Electrochemically generated polymeric films based on natural phenolic antioxidants have been successfully used as a platform for the further immobilization of other metal and metal-containing nanomaterials via electrodeposition. The polymeric coverage of the porous structure increases the surface area of the electrode, leading to a higher loading of the nanomaterial deposited on further steps, as well as improving its distribution on the electrode surface. Moreover, the synergetic effect of both modifiers—in particular, the high electroconductivity and electrocatalytic effect–is observed in the case of such a modification.

The combination of electropolymerized natural phenolic antioxidants with metal nanoparticles provides their uniform distribution on the electrode surface, and even some regularity in arrangement [89,90,91]. On the other hand, polyphenols can chelate (absorb) metal ions undergoing further electroreduction, and can result in a slow mass transition. The smaller diameter and better size distribution of metal nanoparticles have been obtained in this case [90,91]. The design of novel voltammetric sensors for biologically important analytes has been performed based on these composites.

**Figure 15:**
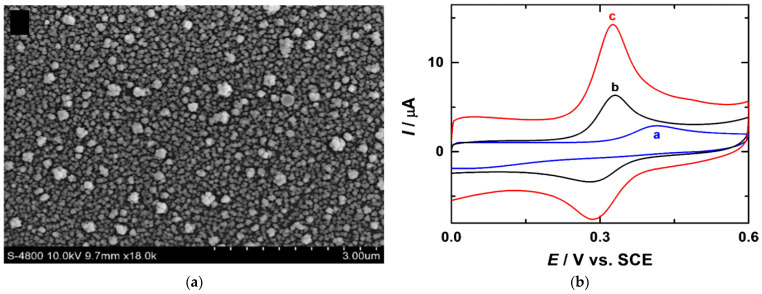
(**a**) SEM image of Au nanoparticles/poly(caffeic acid)/GCE; (**b**) cyclic voltammograms of 50 μM acetaminophen at bare GCE (curve a), poly(caffeic acid)/GCE (curve b), and Au nanoparticles/poly(caffeic acid)/GCE (curve c) in 0.01 M phosphate buffered saline pH 7.4. Reprinted from [89] with permission from Elsevier.

A sensor modified with poly(caffeic acid) and Au nanoparticles has been created using layer-by-layer potentiostatic electrodeposition in a phosphate buffer of pH 7.4 [89]. Firstly, poly(caffeic acid) coverage has been obtained from 0.02 mM caffeic acid at +1.9 V for 100 s. Then, Au nanoparticles have been deposited at −0.6 V for 60 s from 1.0 mM HAuCl_4_. Au nanoparticles have a cluster-like morphology with an approximate particle diameter of 50–200 nm, and are uniformly distributed on the polymeric film surface (Figure 15a). The synergetic effect of poly(caffeic acid) and Au nanoparticles accelerates the electron transfer and provides an electrocatalytic effect for acetaminophen oxidation in a neutral medium (Figure 15b). The sensor shows a linear response to acetaminophen in the range of 0.2–20 and 50–1000 µM with the detection limit of 14 nM. The analytical characteristics are significantly improved in comparison to other electrodes based on Au nanoparticles in combination with graphene [92] or with single-walled carbon nanotubes and co-deposited poly(glutamic acid) [93]. The selectivity of the sensor to acetaminophen has been proven in the presence of inorganic ions, glucose, ascorbic acid, and dopamine. The interference effect of uric acid, starting from a two-fold excess, can be overcome by 15–20-fold sample dilution. The sensor is applicable in the analysis of mouse blood, human urine, and tablets [89].

A paraffin-impregnated graphite electrode modified with a silver nanoparticle included in the polyrutin film has been developed [90]. The electrode acts as a sensor for tyrosine and tryptophan. The polyrutin film has been formed by chronoamperometry at 1.4 V for 1 h. Then, a 40 min sorption and chelation of silver ions have been carried out, with a further electrodeposition of silver nanoparticles using potentiodynamic electrolysis from −1.0 to 1.0 V in 0.1 M LiNO_3_. The formation of arrays of silver nanoparticles with an average diameter of 16–23 nm confirms the structuration of the sensor surface. The individual electrocatalytic determination of tyrosine and tryptophan in the ranges of 0.30–10 and 0.7–70 µM with the detection limits of 0.07 and 0.1 µM, respectively, is possible. Unfortunately, the oxidation potentials of tyrosine and tryptophan overlap, allowing for only their total contents evaluation. Another disadvantage of the sensor is its insufficient selectivity as long as a 10-, 50-, and 50-fold excess of ascorbic acid, epinephrine, and serotonin, respectively, show a positive interference effect, limiting the practical application of the sensor.

A similar method is used for the fabrication of a sensor based on the nano-silver-coated polyquercetin composite in combination with other nanomaterials [91]. The complicated and tedious procedure of the layer-by-layer modification of the platinum electrode is used, and includes the following steps:

- The formation of a positively charged choline layer on the bare platinum electrode using cyclic voltammetry in a 1.0 mM choline solution using 0.01 M LiClO_4_ as supporting electrolyte;
- The drop-casting of carboxylated-by-acid treatment MWCNTs via electrostatic adsorption and evaporation of the solvent to dryness;
- The potentiodynamic electropolymerization of quercetin from its 1 mM solution in a 0.1 M phosphate-buffered saline (pH 7.0);
- The chelation and adsorption of silver ions on the polyquercetin surface from its 1.0 mM solution in 0.1 M LiNO_3_ for 30 min;
- The electrodeposition of silver nanoparticles by the voltammetric reduction in 0.1 M LiNO_3_.

Such an approach provides uniformly dispersed silver nanoparticles that are 10 ± 6 nm in diameter. Polyquercetin plays an important role in this process. The sensor has shown significant electrocatalytic activity towards L-cysteine and has been applied for its determination using stripping chronopotentiometry. The accumulation of L-cysteine at 0.5 V for 60 s, with a further registration of the reduction current at 0.13 V, provides its quantification in the range of 0.10–500 nM with the detection limit of 0.030 nM. These characteristics are one of the best ones achieved using electrochemical methods. However, the low selectivity in the presence of tyrosine, tryptophan, glutathione, homocysteine, dopamine, norepinephrine, adrenalin, and ascorbic and uric acids significantly affects the potential practical application of the sensor [91].

Further development in the sensors under consideration is demonstrated with the example of cobalt phosphide nanoparticle electrodeposition [94]. A step-by-step electrosynthesis of a sensor-sensitive layer is based on the preliminary activation of the bare GCE surface by potential cycling in 0.5 M H_2_SO_4_, with the following electrodeposition of poly(caffeic acid) by chronoamperometry at 1.9 V for 150 s in a 20 µM monomer solution in a neutral medium. The electrodeposition of cobalt phosphide nanoparticles has been performed by 15-fold potential cycling from −0.9 to 0 V at a scan rate of 50 mV s^−1^ from 0.05 M Co(CH_3_COO)_2_ and 0.1 M NaH_2_PO_2_ in an acetate buffer of pH 5.0. As confirmed by scanning electron microscopy, the polymer is fully covered by an abundant number of cobalt phosphide particles with a size of less than 200 nm. A significant improvement of the acetaminophen response in comparison to activated GCE, GCE modified with poly(caffeic acid), or GCE modified with cobalt phosphide nanoparticles, indicates the synergetic effect of both modifiers. The sensor allows for the quantification of 0.05–50 µM of acetaminophen with the detection limit of 10 nM. Furthermore, a 100-fold higher concentration of SO_4_^2−^, Cl^−^, NO_3_^−^, L-lysine, glycine, uric, folic, L-ascorbic acids, pyridoxine hydrochloride, and 11 mM of glucose do not show an interference effect on the acetaminophen response, allowing for the practical application of the sensor in bioanalysis. The sensor applicability has been demonstrated for pharmaceutical dosage forms and an evaluation of the antioxidant effect of acetaminophen against the oxidative injury of vascular endothelial cells [94].

**Figure 16:**
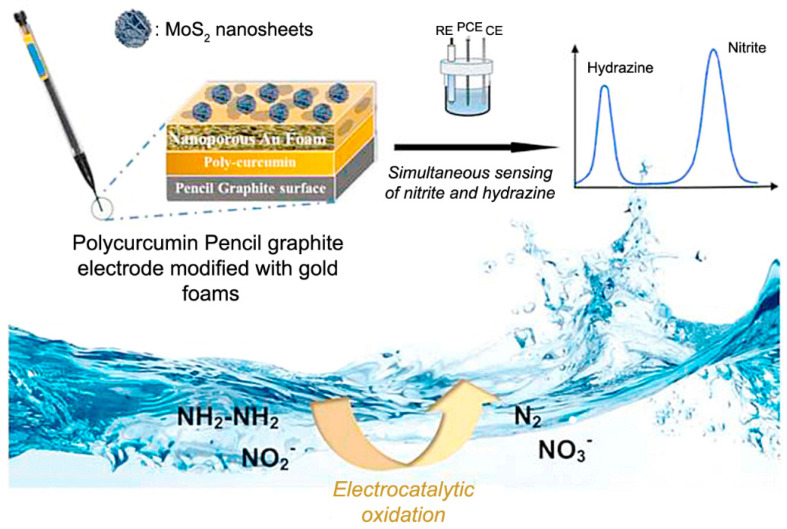
Schematic representation of the sensor based on the polycurcumin/gold foam decorated by molybdenum disulfide nanosheets and analytical application. Reprinted from [95] with permission from Elsevier.

An amperometric sensor based on a pencil graphite electrode covered with electropolymerized curcumin, which is loaded with gold foam decorated by molybdenum disulfide nanosheets, has been constructed for the determination of nitrite and hydrazine in water samples [95]. The scheme of the sensor construction and action is presented in Figure 16. The polycurcumin layer has been electrodeposited by a known protocol [86]. After that, the gold foam has been formed by chronoamperometry at −0.7 V for 60 s in 1.2 mM HAuCl_4_·3H_2_O in 2.4 M H_2_SO_4_. MoS_2_ nanosheets have been electrodeposited on the gold foam surface chronoamperometrically at −1.0 V for 60 s in 5 mM (NH_4_)_2_MoS_4_ in 0.1 M KCl. The sensor responds to nitrite and hydrazine at 0.2 and 0.75 V, allowing for their simultaneous quantification in the ranges of 20–350 and 400–1200 μM for hydrazine and 20–200 and 250–1200 μM for nitrite, with the detection limits of 18.3 and 21.7 nM, respectively. The long-term stability of the sensor response for 30 days has been shown.

Thus, coatings of electropolymerized natural phenolic antioxidants could be considered as perspective substrates for the immobilization of electroactive nanomaterials including the electrogenerated ones. Nevertheless, the number of investigations in this field is quite limited.

## 6. Molecularly Imprinted Polymers Based on the Electropolymerized Natural Phenolics as a Sensitive Layer of Electrochemical Sensors

Sensors based on molecularly imprinted polymers (MIPs) are widely developed devices to date. A high selectivity of the target analytes determination using such sensors is especially demanded in bio-, food, and environmental analysis. The electrochemically synthesized MIPs are of interest. Nevertheless, electrosynthesis is seldom used compared to chemical free radical polymerization, and was recently discussed in [96]. The major advantages of electropolymerization, such as its superior retention of the polymer on the electrode surface, rapidity and simplicity of polymer formation, and possibility to control the thickness and morphology of the polymeric coverage, as well as the exclusion of an additional reagent and toxic organic solvents usage, stipulate the further development of the electrosynthesized MIP-based sensors.

Among the possible functional monomers used, natural phenolic antioxidants have shown potential in the creation of MIPs as recognition elements of electrochemical sensors. Nevertheless, this topic is less studied in comparison to those discussed in Section 3, Section 4 and Section 5, although it is of practical interest.

### 6.1. Protein Imprinted Polymers

The first experiments in the field of MIPs based on the electropolymerized natural phenolics have been presented on protein-imprinted materials using poly(caffeic acid) electrodeposited at the surface of screen-printed carbon electrodes [97,98]. Annexin A3 and microseminoprotein-beta, being biomarkers of prostate cancer, have been used as a template. The imprinted layer of poly(caffeic acid) has been obtained by the drop-casting of 30 μL of a solution containing 0.20 mM of caffeic acid and 5.0 × 10^−3^ ng mL^−1^ of Annexin A3 [97] or 5.0 × 10^−3^ mg mL^−1^ of microseminoprotein-beta [98] in a phosphate buffer of pH 7.2 over the three-electrode system. Then, the constant potential of +2.0 V for 30 s has been applied to the working electrode and the polymerization process has been performed. The polymer-imprinted electrode has been thoroughly washed with ultra-pure water, dried under N_2_, and kept overnight in a 1 M H_2_SO_4_ solution at 45 °C for protein removal. After washing with the phosphate buffer and rinsing with ultra-pure water, with further drying taking place under N_2_, the MIP-based sensors have been ready to use. Both sensors show enough selectivity to target proteins in the presence of biological fluids (synthetic urine and serum). Moreover, sensors demonstrate a stable response within time and can be re-used three times after cleaning with 1 M H_2_SO_4_ for 12 h at 45 °C, and can subsequently be washed with the phosphate buffer and ultra-pure water.

**Figure 17:**
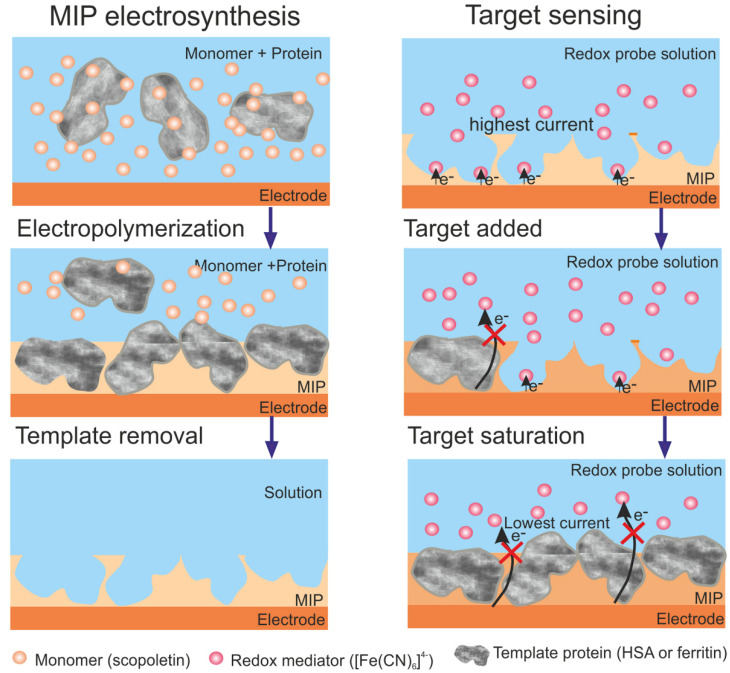
Schematic illustration of the electrosynthesis of surface-imprinted polyscopoletin nanofilm on a gold electrode surface and its use for the detection of the target proteins. Reprinted from [99] with permission from Elsevier.

Another functional monomer recently used for the fabrication of protein-imprinted polymers is scopoletin (a natural phenolic compound of the coumarins group). Similar to flavonoids, it is electropolymerized with the formation of insulating polymeric films [99]. The ability to entrap proteins during the electropolymerization process results in MIP formation (Figure 17). A wide range of proteins have been used as a template for the fabrication of MIPs-based sensors; in particular, human serum albumin and ferritin [99], laccase [100], cytochrome P450 BM3 [101], transferrin [102], cytochrome c [103], and hexameric tyrosine-coordinated heme protein [104].

Various approaches are used for the electrosynthesis of protein-imprinted polyscopoletin:

- Electrodeposition on the gold electrode surface from the solution containing scopoletin and protein template using the potentiodynamic mode [99,100];
- Electrodeposition on the gold or fluorine-doped indium tin oxide after preliminary incubation in the protein template solution, followed by scopoletin electropolymerization in the solution using repeated chronoamperometry by applying multiple alternating oxidation and reduction pulses of 1 s at 0.9 V and 5 s at 0 V [101,102];
- Electrodeposition on the gold electrode covered with a self-assembled monolayer of mercaptoundecanoic acid from the solution containing scopoletin and a protein template by single redox potential pulses at 0 V for 15 s and 0.5 V for 35 s [103];
- Electrodeposition on the gold electrode covered with a self-assembled monolayer of mercaptoundecanoic acid after preliminary incubation in the protein template solution, followed by scopoletin electropolymerization in a solution using a single potential pulse at 0.7 V for 5 s and 0 V for 5 s [104].

The template removal from the MIPs created is based on various treatments depending on the nature of the protein and its properties. In some cases, a simple incubation in solvents (NaOH [100], imidazole in 100 mM sodium phosphate pH 7.4 [101] or H_2_SO_4_ [103]) for a certain time (from 90 min [103] to overnight [100,101]) at a given temperature provides a successful removal of the protein template. A combination of sensor incubation in 50 mM glycine-HCl (pH 2.2) with shaking at 300 rpm for 1 h (25 °C) is required for the removal of hexameric tyrosine-coordinated heme protein [104]. More tedious procedures of template removals are based on the consecutively immersing of the MIP-based sensor in 5 mL of gently agitated solutions of 5 mM NaOH (10 min), 5% SDS (5 min), 5 mM NaOH (10 min) for human serum albumin, and, additionally, in 0.05% Tween 20 (5 min), 5 mM NaOH (10 min) for ferritin, and, finally, in deionized water for 5 min in both cases [99]. The enzymatic digestion with proteinase K under shaking conditions at 300 rpm for 4 h with further washing by overnight shaking in phosphate-buffered saline provides a full removal of transferrin [102].

The protein-imprinted sensors based on poly(caffeic acid) and polyscopoletin do not show their own electrochemical activity, excluding cytochrome c [103] and hexameric tyrosine-coordinated heme protein [104]-imprinted polymers. Therefore, redox-active probes, such as ferri- [101] and ferrocyanide ions [99], or their mixture [97,98,102], as well as catechol [100], are used as voltammetric signal-forming substances. The sensor response is affected by protein binding to the available sites in the polymeric network. The changes in the redox probe response are proportional to the protein concentration. In the case of cytochrome c [103] and hexameric tyrosine-coordinated heme protein [104]-imprinted polymers, the sensor response is based on the direct electron transfer used in electrocatalysis.

The sensors developed allow for an evaluation of the laccase activity [100], recognition and discrimination of domains of cytochrome P450 BM3 [101], elucidation of the transferrin binding behavior [102], study of the direct electron transfer of cytochrome c [103], and the bioelectrocatalytic reduction of hydrogen peroxide using hexameric tyrosine-coordinated heme protein-imprinted polymer [104]. The analytical application of the protein-imprinted sensors is shown for the annexin A3, microseminoprotein-beta, human serum albumin, and ferritin (the corresponding analytical characteristics are presented in Table 4).

### 6.2. Low-Molecular Organic Compounds Imprinted Polymers

**Figure 18:**
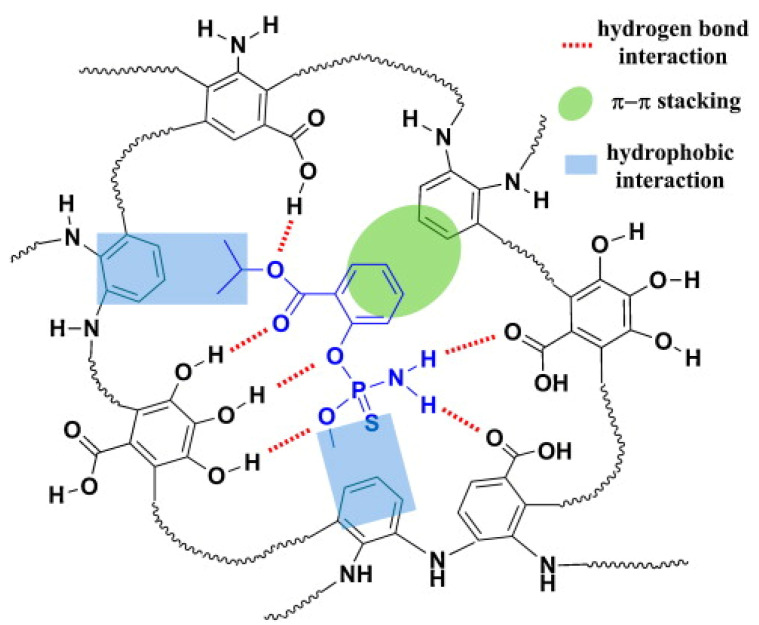
Schematic representation of the specific recognition of isocarbophos by a functionalized cavity of the terpolymer matrix based on poly(*o*-phenylenediamine-co-gallic acid-co-*m*-aminobenzoic acid). Reprinted from [105] with permission from Elsevier.

The preparation of the MIPs based on natural phenolic antioxidants for the binding of low-molecular organic compounds requires several functional monomers. This is caused by the necessity to form active binding centers in the final polymer structure showing an affinity to the template. Depending on the template structure, H-bonding, hydrophobic interactions (including π-π staking), or their complex can be realized (Figure 18) [105]. A combination of these interactions can provide more selective binding. Another factor to be taken into account is the porous structure of the polymer, including the formation of a cross-like structure [106] or terpolymer cavities [105,107] that create a microenvironment for the successful recognition of the target molecule. Thus, the size, shape, and distribution of the functionalities of the imprinting cavities generated by the copolymerization of the adequate combination of several different monomers match better with the template than that formed by the single monomer [106].

The MIP-based sensors created have shown a high sensitivity and sufficient selectivity towards target analytes. The analytical characteristics achieved are summarized in Table 5. Gallic acid [105,106,107] and quercetin [108,109] are used as one of the functional monomers for MIP fabrication.

The electrochemical co-polymerization of gallic acid or quercetin with other functional monomers is performed in the potentiodynamic mode [105,106,107,108,109]. The conditions of the MIP electrodeposition are optimized in each case depending on the template and functional monomer properties. Thus, 10 mM solutions of gallic acid and *o*-phenylenediamine and 1 mM of melamine in a phosphate buffer of pH 4.5 containing 0.1 M KCl and 20-fold potential cycling from −0.8 to 1.2 V at the scan rate of 50 mV s^−1^ have been applied for the creation of a MIP-based sensor to melamine [106]. The fabrication of sensors based on the molecularly imprinted terpolymers of poly(*o*-phenylenediamine-co-gallic acid-co-*m*-aminobenzoic acid) can be performed from 40 µM of *o*-phenylenediamine, 20 µM of gallic acid, 20 µM of *m*-aminobenzoic acid, and 10 µM of templates (isocarbophos or mesalamine) by 15 potential scans from −0.4 to 0.8 V at a scan rate of 50 mV s^−1^ in a phosphate buffer (pH 2.0 or 4.0) containing 0.2 M KCl [105,107]. The additional potentiostatic electrodeposition of silver nanodendrites on the surface of a mesalamine-imprinted polymer has been used for the amplification of the electrochemical response of the sensor [107]. A GCE preliminary modified with gold nanoparticles by the potentiostatic reduction of HAuCl_4_ has been used as a platform for the further electrosynthesis of MIP based on poly(quercetin-co-resorcinol) [108,109]. An electroplating of MIP has been performed in an acetate buffer (pH 6.0 [108] or 5.8 [109]) containing 5 mM of quercetin, 2 mM of resorcinol, and 1 mM of the template by eight [108] or eleven [109] cycles in the range of −0.2 to 0.9 V with a scan rate of 50 mV s^−1^ [108,109]. Moreover, additional defects in the obtained MIP layer have been covered with dodecanethiol by the incubation of the electrode in a 0.05 M dodecanethiol ethanolic solution for 10 [108] or 24 h [109].

Template removal for these sensors is relatively rapid and simple. An immersion of the electrode to the appropriate solvent (ethanol–water (2:1 *v*/*v*) [106,108], NaOH in 50% (*v*/*v*) acetonitrile [106], or 0.8% ethanol acid solution [108,109]) for a certain time (10 s–10 min) provides a full removal of the target molecule.

The analytical signal of the sensors depends on the electroactivity of the target analyte. In the case of electrochemically inert isocarbophos [105] and melamine [106], the changes in the reduction current of hexacyanoferrate(III) ions under conditions of a differential pulse [105] or cyclic [106] voltammetry are used as the analytical signal of the sensor. The own response of mesalamine and methyl parathion bound at the electrode surface is used for analytical purposes [107,108]. The response of the capacitive MIP sensor to methyl parathion is based on the changes in double-layer capacitance in impedimetric measurements in the presence of a 5 mM mixture of hexacyanoferrate(II)/(III) ions as a redox probe containing 0.1 M NaClO_4_ as the supporting electrolyte [109]. The rebinding of the methyl parathion leads to the formation of a compact film at the sensor surface that hinders the electron transfer of the redox probe, i.e., the increase in the capacitance.

Sensors show a selectivity to the target analytes in the presence of typical interfering compounds, as well as structurally related compounds of the same group. Thus, tyrosine and phenylalanine have almost no interference (<1.5%), histidine shows only a slight interference, and glycine has a slightly greater effect (4.02%) on the melamine response [106]. Such behavior is caused by the molecular size of the interferences affecting the penetration ability to the recognition sites of the MIP film. In the case of mesalamine, the selectivity of the sensor in the presence of minoxidil, warfarin, and phenylephrine has been proven [107]. The excellent selectivity of the MIP-based sensors towards organophosphate pesticides (isocarbophos [105] and methyl parathion [108,109]) has been achieved in the presence of trichlorfon, chlorpyrifos, and methamidophos [105], as well as fenitrothion and imidacloprid [108], phoxim, malathoate, omethoate, and (2,4-dichlorophenoxy) acetic acid [109].

The high sensitivity and selectivity of the MIP-based sensors under consideration allow for their application in real sample analysis, as has been demonstrated for the quantification of isocarbophos in cabbage and cowpea samples [105], melamine in milk [106], mesalamine in spiked human serum and urine [107], and methyl parathion in spiked water, tangerine, and sweet potato leaves juices [108], as well as in different water samples and fruit surfaces [109].

## 7. Problems in the Field of Electrochemical Sensors Based on Electropolymerized Natural Phenolic Antioxidants

Natural phenolic antioxidants form self-limiting, non-conducting polymeric coverages during electropolymerization. The polymers obtained show permselective membrane properties, a high sensitivity, fast response, and sufficient reproducibility, making them attractive for the design of the electrochemical sensor. Nevertheless, there are several unsolved problems to date.

The chemical structure of electrosynthesized polymers is out of consideration in the majority of investigations. This is caused by the technical difficulties in performing a chemical characterization via Fourier transform infrared or NMR spectroscopy as long as polymeric coverage is obtained via electrolysis and fixed at the electrode surface. More complex problems exist in the case of the combination of several modifiers, such as carbon nanotubes, metal nanoparticles, etc. The application of molecular design methods and organic electrosynthesis can likely be helpful for this purpose.

The choice of polymer and conditions of its preparation is another actual aspect to be considered. The optimization of natural phenolic antioxidant electropolymerization conditions, i.e., the structure and properties of the final coverage, have to be studied in each case in order to provide the best response of the sensor towards the target analyte. A high-throughput synthesis and electrochemical screening of a library of modified electrodes could be an effective approach for the realization of this idea. The combinatorial method is based on the covalent attachment of the substrate to the electrode surface through a linker, and after several synthetic steps, the product is generally cleaved from the solid support and screened for the properties of interest [110].

Although many electrochemical sensors based on the electropolymerized natural phenolic antioxidants were developed, the stability of their response is often ignored. Therefore, the commercial production and application of the sensors in real practice are questionable. The efforts have to be focused on the creation of stable coverages that are able to be stored at least several months after preparation without signal loss. Another way to solve this problem is the application of a simple protocol of electropolymerization that can be easily realized in the laboratory.

Thus, further investigations and developments in the field of electrochemical sensors based on electropolymerized natural phenolic antioxidants are required.

## 8. Conclusions

Electropolymerized natural phenolic antioxidants are prospective materials for the creation of electrochemical sensors. A great variety of these compounds of different classes and chemical structures with a common organic skeleton open a wide area of investigations.

The electropolymerization of natural phenolic antioxidants leads to the formation of insulating polymers. Polymer-based sensors show that low capacitive currents significantly improve the shape of target analyte voltammograms and the accuracy of their determination. The electrochemical properties of the final polymeric coverage strongly depend on the type of monomer, as well as on the conditions of electropolymerization. Phenolic antioxidants containing catechol fragments in their structure form electroactive polymeric layers that are able to act as redox-mediators, providing an electrocatalytic effect on the oxidation of organic compounds. On the contrary, monophenolic antioxidants, such as *p*-coumaric acid, form electrochemically inactive polymeric coverage. The electropolymerization conditions have to be optimized in order to obtain the best response of the target analyte.

The insufficient conductivity of the polymers based on the electropolymerized natural phenolic antioxidants can be improved by their combination with conductive materials, such as carbon nanomaterials or metal/metal oxide nanoparticles. Such an approach positively affects both the electroconductivity and the effective surface area of the sensor surface on which polymer electrodeposition is performed. The combination of modifying layers provides an increase in the sensitivity and selectivity of the target analyte response. Furthermore, screen-printed electrodes with predeposited carbon nanomaterials or metal/metal oxide nanoparticles can be used for the immobilization of polymeric coverage, which will be useful for routine analysis. This can significantly shorten the sensor preparation time, and, consequently, the whole analysis.

The porous structure of the polymers obtained provides various properties, allowing for their application as a sensitive layer of the sensors that gives a response to a wide range of both inorganic and organic compounds. The polymeric films can act as a semipermeable membrane, allowing for the creation of electrochemical sensors for low-molecular-weight compounds, such as hydrogen peroxide, nitric oxide, and ascorbic acid. The presence of electron-donating atoms in the polymeric film structure makes them able to bind heavy metals, providing their preconcentration at the sensor surface. In the case of organic analytes, a porous structure of the polymeric coverage is capable of adsorbing analytes due to the structural similarity and size effects, which stipulate both the individual or group selectivity of the sensor created and their wide application area in electroanalysis. The determination of natural phenolic antioxidants and total antioxidant parameters is of special interest and has significant practical potential. The use of the own response of antioxidants at the sensors described excludes the use of auxiliary reagents, which is typical for standard methods of antioxidant property evaluation. The possibility of the application of natural phenolic antioxidants as co-monomers in the creation of electrochemically synthesized MIPs is successfully confirmed, although there is a lack of investigation in this field.

Future development of the electrochemical sensors based on the electropolymerized natural phenolic antioxidants can be considered in the following directions:

- An investigation of the polymeric coverage structure and clarification of the electropolymerization reaction mechanisms and schemes;
- An application of other nanomaterials (metal and metal oxide nanoparticles, other nanostructured compounds, etc.) as a platform for the electrodeposition of natural phenolics based polymers;
- The development of the electrochemical sensors based on the copolymerization of natural phenolic antioxidants of the same or different classes;
- The further development of the electrochemical sensors using electropolymerized natural phenolic antioxidants as a platform for the immobilization of other modifiers;
- The creation of the sensors based on the electrochemically synthesized MIPs for the low molecular biologically active compounds, other than organophosphate pesticides and melamine;
- The application of mathematical design methods and machine learning for the optimization of electropolymerization conditions and the choice of monomers for sensor creation, including MIPs-based ones;
- The fabrication of sensors characterized by a high long-term stability of the response, allowing for their commercial production, storage, and application in real practice.

The high diversity of natural phenolic antioxidants and their ability to electro-oxidate and polymerize at the electrode surface leads us to expect an increase in the interest towards the electrochemical sensors based on this type of modifier in the following years. The application trend of the sensors to biomedicine, environment, and food control will be maintained. Attention could be paid to the estimation of total parameters using the group selectivity of the sensors under consideration.

## Figures and Tables

**Table 1:** Electropolymerization conditions of natural phenolics in potentiodynamic mode.

Monomer	Electrode	*c*_monomer_	Supporting Electrolyte	Polarization Window (V)	υ (mV s^−1^)	Number of Scans	Refs.
Eugenol	Pt			0.0–2.2	100	10	[21]
				0.0–0.70	5	10	[22]
	GCE			0.0–2.0	100	10	[23]
	Au	10 mM	0.1 M NaOH	−1.0–1.0	50	5	[24]
	GCE			−1.0–0.60	100	10	[24]
	Au			0.0–0.70	100	20	[25]
	Ti			−0.60–1.5	10	2	[26]
*p*-Coumaric acid	Graphite electrode	2.5 mM	0.1 M NaOH	−0.25–0.8	50	25	[37]
Rutin	Paraffin-impregnated graphite electrode	1.0 mM	0.1 M phosphate buffer pH 7.0	0.0–1.4	50	50	[38]
	CPE	1.0 mM	0.1 M phosphate buffer pH 7.0	−0.8–1.2	50	30	[39]
Vanillin	CPE	1.0 mM	0.01 M NaOH	−0.40–1.2	100	10	[40]
		10 μM	0.1 M NaOH	−0.40–1.2	50	15	[41]
Tannin	CPE	1.0 mM	0.01 M NaOH	−0.40–1.2	100	10	[42]
Curcumin	Electroactivated GCE	50 μM	0.1 M phosphate buffer pH 8.0	0.15–0.55	100	16	[43]
Catechin	Activated CPE	1.0 mM	0.1 M phosphate buffer pH 7.4	0.20–1.6	50	15	[44]
Luteolin Kaempferol	GCE	1.0 mM	0.1 M phosphate buffer pH 7.5	0.10–0.90	100	30	[45,46]
Apigenin	GCE	1.0 mM	0.1 M phosphate buffer pH 7.0	0.0–1.4	100	30	[47]

**Table 2:** Analytical characteristics of neuromediator determination using sensors based on the electropolymerized natural phenolic antioxidants.

Sensor	Method	Analyte	Linear Dynamic Range (µM)	Detection Limit (µM)	Refs.
Polyeugenol/Pt	DPV ^1^	Dopamine	0.1–50	0.1	[21]
Polyrutin/PIGE ^2^	DPV	Epinephrine	3.0–90.0	0.8	[38]
		Serotonin	0.3–9.0	0.1	
		Ascorbic acid	2.0–60.0	1.0	
Polyvanillin/CPE	Cyclic voltammetry	Dopamine	1000–3000	-	[40]
		Epinephrine	10–60	5.4	[41]
		Uric acid	10–60	5.40	
Polytannin/CPE	Cyclic voltammetry	Dopamine	1000–3500	-	[42]
Polycurcumin/GCE	Linear sweep voltammetry	Epinephrine	4.97–230.76	0.054	[43]
		*p*-Acetoaminophenol	0.99–230.76		
Polycatechin/CPE	Amperometry	Dopamine	0.010–0.78	0.0005	[44]
		Serotonin	0.030–2.34	0.003	
Poly(caffeic acid)/GCE	CV	Dopamine	1.0–40	0.40	[49]
		Ascorbic acid	20–1200	9.0	
	DPV	L-DOPA	1.0–50	0.14	[51]

^1^ Differential pulse voltammetry. ^2^ Paraffin-impregnated graphite disk electrode.

**Table 3:** Analytical capabilities of the sensors based on the carbon nanomaterials and electropolymerized phenolic antioxidants.

Electrode	Sensitive Layer	Method	Analyte	Detection Limit (µM)	Linear Dynamic Range (µM)	Sample	Refs.
GCE/MWCNTs	Poly(vanillic acid)	Amperometry				Urine	[33]
		0.100 V	Ascorbic acid	3.5	5–120		
		0.225 V	Dopamine	1.5	5–120		
		0.325 V	Uric acid	4.5	5–120		
	Poly(ferulic acid)	Amperometry 0.2 V	NADH	17.73	59.1–1560	Pharmaceutical dosage forms	[34]
			Dopamine	2.210	5.00–120.0		
			Epinephrine	22.28	73.0–1406		
	Poly(*p*-coumaric acid)	DPV	L-cysteine	1.1	7.5–50; 50–1000	Human urine	[35]
	Poly(gallic acid)	DPV	Quercetin	0.054	0.075–25; 25–100	Medicinal herbs	[32]
		AdASWV ^1^	Gallic acid	3.22	4.97–33.8	Model solutions	[52]
		Chronocoulometry 1.0 V	Quercetin	0.0029	0.010–0.25; 0.25–250	Medicinal plant tinctures	[73]
		DPV	Capsaicin	0.0029	0.010–1.0; 1.0–50	Red hot pepper spices and *Capsicum annuum* L. tinctures	[74]
			Dihydrocapsaicin	0.0059	0.025–0.75; 0.75–75		
			Nonivamide	0.0061	0.025–5.0; 5.0–75		
	Poly(ellagic acid)	DPV	Naringin	0.014	0.050–1.0; 1.0–100	Grapefruit juices	[75]
	Polyeugenol	DPV	Catechin	0.21	1.00–250	Antioxidant capacity of wine	[56]
	Polyquercetin	DPV	Gallic acid	0.10	0.50–10; 10–750	Antioxidant capacity of tea	[76,77]
			Catechin	0.024	0.10–10; 10–250		
			Epigallocatechin gallate	0.014	0.050–10; 10–100		
		Chronoamperometry 0.2 V	Gallic acid	0.063	0.25–750	Antioxidant capacity of tea	[78]
	Polycurcumin	Amperometry 0.25 V	Hydrazine	1.4	2–44	-	[79]
PIGE/MWCNTs	Polycurcumin	CV	Butylated hydroxyanisole	0.23	3.37–332	-	[80]
GCE/Carboxylated MWCNTs	Polyquercetin	DPV	L-DOPA	0.381	0.90–85.0	Biosamples and pharmaceutical dosage forms	[81]
			Uric acid	0.575	1.0–125		
			Tyramine	0.647	0.70–75		
GCE/CNTs ^2^	Poly(caffeic acid)	CV	Glutathione	0.5	50–5000	-	[82]
GCE/Carbon black	Poly(caffeic acid)	Amperometry 0.25 V	NADH	3.7	-	-	[83]
GCE/Highly conductive carbon black	Poly(syringic acid)	Chronoamperometry 0.04 V	L-cysteine	0.639	20–100; 100–1000	Simulated blood serum and chicken samples	[84]

^1^ Adsorptive anodic square wave voltammetry. ^2^ Carbon nanotubes.

**Table 4:** Figures of merits of the sensors with protein-imprinted polymers based on the natural phenolic antioxidants.

Functional Monomer	Template/Analyte	Electrode Material	Detection Method	Detection Limit	Linear Dynamic Range	Refs.
Caffeic acid	Annexin A3	Screen-printed carbon electrode	SWV	0.095 ng mL^−1^	0.10–200 ng mL^−1^	[97]
	Microseminoprotein-beta		SWV	0.12 ng mL^−1^	0.50–100 ng mL^−1^	[98]
Scopoletin	Human serum albumin	Au	CV	3.7 mg L^−1^	20–100 mg L^−1^	[99]
	Ferritin			10.7 mg L^−1^	120–360 mg L^−1^	

**Table 5:** Analytical characteristics of the imprinted electropolymerized natural phenolic antioxidant-based sensors for low-molecular organic compounds.

Functional Monomer	Template/Analyte	Electrode Material	Detection Method	Detection Limit	Linear Dynamic Range	Refs.
Gallic acid + *o*-phenylenediamine	Melamine	GCE	SWV	1.4 nM	5.0–100.0 nM	[106]
Gallic acid + *o*-phenylenediamine + *m*-aminobenzoic acid	Isocarbophos	GCE	DPV	0.0201 µM	0.075–50; 50–100 µM	[105]
Gallic acid + *o*-phenylenediamine + *m*-aminobenzoic acid (and further electrodeposited Ag nanodendrites)	Mesalamine	GCE	Anodic stripping SWV	0.015 µM	0.05–100 µM	[107]
Quercetin + resorcinol	Methyl parathion	Au nanoparticles/GCE	CV	0.01 μM	0.05–15 µM	[108]
			EIS ^1^	0.34 nM	70–1000 nM	[109]

^1^ Electrochemical impedance spectroscopy.

## Data Availability

Not applicable.
